# *In vivo* and *in vitro* recombinant systems of a novel variant demonstrate cross-reactive neutralization for the HCV model virus, Norway rat hepacivirus

**DOI:** 10.1371/journal.ppat.1013127

**Published:** 2025-09-25

**Authors:** Caroline E. Thorselius, Andreas Kok, Raphael Wolfisberg, Ulrik Fahnøe, Matthew J. Kennedy, Emma A. Lundsgaard, Lotte Mikkelsen, Mads K. Larsen, Satyapramod Murthy, Sheetal Trivedi, Amit Kapoor, Troels K. H. Scheel, Kenn Holmbeck, Jens Bukh

**Affiliations:** 1 Copenhagen Hepatitis C Program (CO-HEP), Department of Infectious Diseases, Copenhagen University Hospital, Hvidovre and Department of Immunology and Microbiology, Faculty of Health and Medical Sciences, University of Copenhagen, Copenhagen, Denmark; 2 Center for Vaccines and Immunity, Research Institute at Nationwide Children’s Hospital, Columbus, Ohio, United States of America; Princeton University, UNITED STATES OF AMERICA

## Abstract

The lack of immunocompetent animal models severely impedes the development of vaccines against hepatitis C virus (HCV) and researchers have therefore explored the application of surrogate virus models. Norway rat hepacivirus 1 (NrHV) is a promising candidate, mirroring HCV genetic structure, pathogenesis, and immunity. However, NrHV experimental tools are limited to a single variant, RHV-rn1, and do not represent the vast genetic heterogeneity of HCV. To increase NrHV utility for HCV vaccine research, we characterized a novel variant and developed recombinant tools to study cross-reactive neutralizing antibody responses. We sequenced the isolate, NrHV-K, and created a molecular consensus clone, pNrHV-K, closely related to the original NrHV prototype strain, NYC-C12. Intrahepatically inoculated RNA-transcripts from pNrHV-K resulted in chronic infection in Lewis rats. Passaging of NrHV-K in severe combined immunodeficiency mice led to persistent infection. However, the mouse-passaged virus did not prolong infection in immunocompetent mice compared with the wild-type NrHV-K variant. Infection in naïve Lewis rats with mouse-passaged virus resulted in a subset of rats clearing the infection during the acute phase, demonstrating a dichotomous infection outcome in inbred rats. We further adapted NrHV-K to efficiently infect rat hepatoma cells and showed that antibodies from RHV-rn1 and NrHV-K infected rats cross-neutralized both variants. The development of additional experimental systems for NrHV variants permits studies addressing the importance of strain diversity. This advancement aids in the quest for multivalent immune responses against diverse NrHV isolates, offering insights into cross-reacting immunity important for future HCV vaccine design.

## Author summary

Vaccine development for hepatitis C virus (HCV) is currently impeded by the lack of suitable animal models. To address this, the Norway rat hepacivirus 1 (NrHV), a virus relatively closely related to HCV, is being explored as a potential model for studying different vaccine strategies. A challenge in this area is the limited number of virus variants available to test how broadly a vaccine can protect. In our study, we characterized a new variant, called NrHV-K, and developed key research tools, including an infectious clone and a cell culture virus system, to enhance its utility for research. Notably, after infecting immunodeficient mice, we discovered that this new variant can be cleared in the acute phase of infection in inbred Lewis rats. This could enable comparisons of immune responses between rats that eliminate the virus and those that develop chronic infections. Additionally, we found that the immune system produces neutralizing antibodies capable of targeting different virus variants. Our findings provide essential tools and insights that could significantly improve the use of NrHV in developing vaccine concepts for HCV.

## Introduction

Chronic hepatitis C virus (HCV) infection causes over 250,000 deaths annually despite the existence of direct-acting antiviral (DAA) therapy with cure rates of ~95% [1,2]. Limited healthcare access in developing countries, inadequate testing strategies, and high costs of DAA treatments expose the limitations of relying solely on DAAs to achieve the World Health Organization’s objective of global HCV elimination [3]. This highlights the urgent need to develop an effective prophylactic HCV vaccine. Despite decades of research, this objective remains elusive, and only a single vaccine candidate has progressed to a phase II clinical trial where prevention of chronic infections was not achieved [4].

One key obstacle in HCV vaccine development is the wide genetic heterogeneity within the eight genotypes and close to 100 subtypes of the virus, which necessitates the development of a vaccine effective against multiple HCV genotypes [3,5]. In chimpanzees, it has been demonstrated that animals that previously cleared a primary infection could, in some instances, more rapidly clear the infection upon rechallenge with a heterologous variant [6–8], suggesting that broad immunological protection is potentially achievable. However, while cross-reactivity of neutralizing antibodies (nAbs) can be assessed *in vitro* and *in vivo* [9–12], exploring broad protection by cellular immunity presents more significant challenges.

Since the only immunocompetent animal model susceptible to HCV infection, the chimpanzee, is no longer available for research, developing an effective HCV vaccine is significantly impeded [13,14]. An alternative approach is to utilize closely related animal hepaciviruses as surrogate models for HCV [15]. Since 2011, new hepaciviruses have been identified in various animal hosts, including horses, monkeys, and rodents [15–17]. Norway rat hepacivirus 1 (NrHV, strain NYC-C12) was discovered in 2014 in a metagenomic survey of *Rattus norvegicus* caught in New York City [18]. We previously determined that another NrHV strain, RHV-rn1, causes chronic infections in laboratory rats and acute resolving infections in laboratory mice [19,20]. The size of the RHV-rn1 genome is 9,656 nucleotides (nt), and like HCV, it contains two binding sites for microRNA 122 (miR-122) in the 5’ untranslated region (UTR) and a single open reading frame (ORF) encoding a polyprotein predicted to be processed into three structural and seven nonstructural proteins [19]. The resemblance of NrHV with HCV, including genome structure, pathogenesis, and induction of similar cellular immune responses, makes it an attractive challenge model [19,21]. To this end, we recently improved the utility of the model by developing an infectious cell culture system for the NrHV isolate RHV-rn1 (RHVcc-1) and the related NrHV-B (NrHV-Bcc), enabling the evaluation of natural and vaccine-induced humoral immunity *in vitro* [22,23].

While NrHV serves as an attractive model for HCV research, there are currently only three closely related isolates of NrHV that have been fully sequenced and used in research studies, RHV-rn1, NrHV-A, and NrHV-B (GenBank number KX905133.1, MF113386.1, and ON758386.1, respectively) [19,20,24]. For the originally characterized isolate, NYC-C12 (NC_025672), only the ORF and the 3’ 99 nt of the 5’UTR have been sequenced, and this strain has not been employed experimentally [18]. This scarcity of experimental systems for different NrHV variants thus limits its use for testing vaccine-induced immunity across an antigenically broad range of viruses.

In this study, we characterized NrHV-K, a variant isolated from a feral rat captured in New York City. We constructed an *in vivo* infectious molecular clone and an infectious cell culture system, drawing upon previous methodologies applied to RHV-rn1 [22,24]. We used these systems to assess cross-reacting nAbs elicited by the two NrHV variants, RHV-rn1 and NrHV-K. Given the prominence of mouse models in immunological research, facilitated by the extensive availability of genetic modifications and research reagents, our study also aimed to assess the viability and adaptation of NrHV-K in mice. This effort was driven by the objective of strengthening the utility of NrHV as a surrogate model for HCV research, thereby contributing to the broader understanding and development of strategies to eliminate HCV [25].

## Results

### Full-length sequence analysis of rat hepacivirus isolate NrHV-K

**Fig 1:**
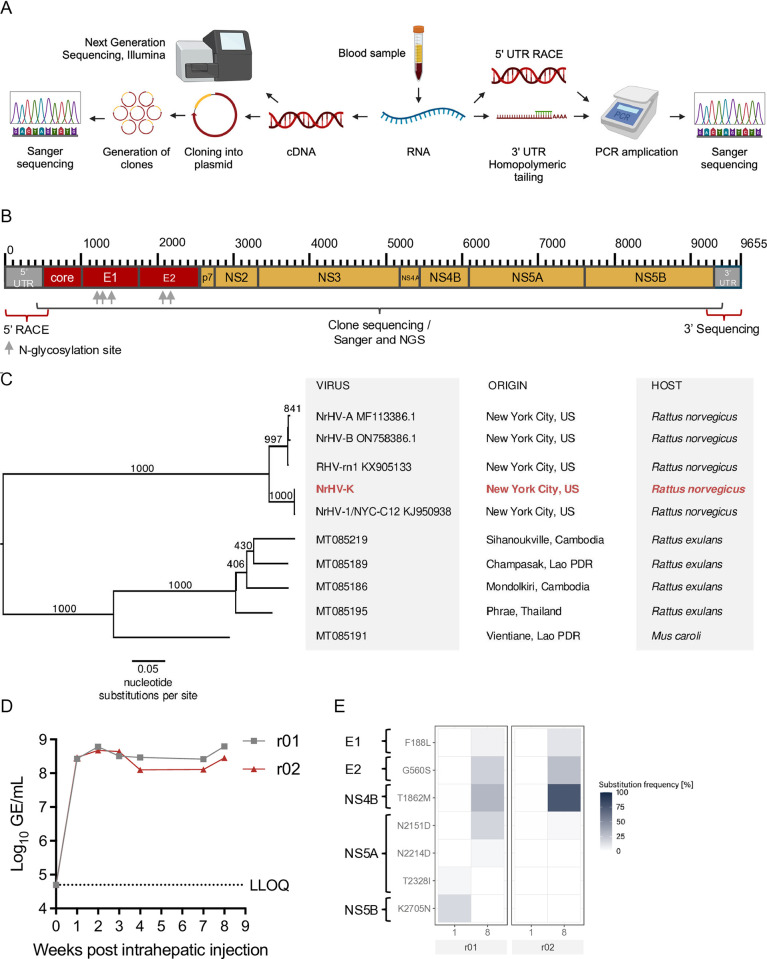
Cloning and sequencing of a rat hepacivirus isolate NrHV-K closely related to the NYC-C12 prototype. (A) Strategy for obtaining the complete NrHV-K sequence using viral RNA extracted from a Lewis rat blood sample following inoculation with serum from a feral *Rattus norvegicus* [18]. Created with BioRender. (B) Genome organization of NrHV-K, grey regions represent untranslated regions (UTRs), red regions structural proteins, and yellow regions nonstructural proteins. Prediction using the RHV-rn1 sequence (GenBank number KX905133.1) [19]. The sequencing strategy is listed for bracketed regions. Grey arrows indicate glycosylation sites predicted by NetNGlyc - 1.0 [26]. (C) Phylogenetic analysis by maximum likelihood phylogenetic tree representation of full-length open reading frame (ORF) nucleotide sequences of rodent hepaciviruses [18,20,27]. GenBank accession numbers with taxon names, sample locations, and host genera are listed in separate columns. Numbers in the tree represent bootstrap support values (1000 replications). (D) Genomic equivalent (GE) titers of NrHV-K in serum collected from two Lewis rats intrahepatically infected with *in vitro* transcribed NrHV-K RNA. The lower level of quantification (LLOQ) was 5x10^4^ GE/mL. Error bars indicate the standard deviation of technical duplicates. (E) Virus evolution analysis of NrHV-K ORFs. Amino acid (aa) position and substitution frequency as % of total reads, using consensus NrHV-K sequence as a reference genome. Weeks-post infection and animal ID (shaded area) are shown below the heat map. The aa substitutions in the figure represent substitution frequencies >3.287% (median error (%) plus three times the standard deviation of the MiSeq Illumina platform [28]) for at least one sample.

Viral RNA extracted from the serum of an experimentally infected Lewis rat [18] was sequenced (Fig 1A). A consensus ORF sequence of 8,877 nt, including the termination codon and an encoded polyprotein of 2,958 amino acids (aa), was identified (S1 and S2 Tables). We moreover determined the length of the 5’ UTR and 3’ UTR to be 484 and 294 nt, respectively, including two predicted miRNA-122 binding sites in the 5’ UTR (S1 Fig), making the total genome length 9,655 nt (Fig 1B). We designated this new isolate NrHV-K (GenBank number PV553238).

Phylogenetic analysis of the complete ORF nt sequence revealed that NrHV-K is closely related to the prototype NrHV isolate NYC-C12 (Fig 1C). Sequence alignment showed only 14 nt and 4 aa positions differing between NrHV-K and NYC-C12 (Tables 1, S3 and S4).

**Table 1:** Number of diverging aa residues and total residue number in NrHV-K, NYC-C12, and RHV-rn1 proteins, respectively, across the entire polyprotein.

	Differences in amino acid residues between NrHV-K and isolates:	
	NYC-C12	RHV-rn1
**Core**	0/ 175	1/ 175
**E1**	1/ 242	9/ 242
**E2**	0/ 272	10/ 272
**p7**	0/ 54	1/ 54
**NS2**	0/ 199	4/ 199
**NS3**	0/ 624	3/ 624
**NS4A**	0/ 54	1/ 54
**NS4B**	2/ 249	1/ 249
**NS5A**	1/506	18/ 506
**NS5B**	0/ 583	5/ 583
**ORF**	**4/ 2,958**	**54/ 2,958**

NrHV-K and RHV-rn1 ORFs showed greater differences at the nt and aa levels, with 524 nt and 54 aa differences, respectively (Tables 1 and S4). NrHV-K retains previously predicted polypeptide cleavage sites and conserved lengths of the resulting proteins described for RHV-rn1 [19]. The analysis, moreover, identified five potential glycosylation sites within the envelope proteins also found in NYC-C12 and RHV-rn1, with three in E1 and two in E2 (Fig 1B).

**Table 2:** Cell culture-adapted viruses generated for this study. Amino acid (aa) residues diverging from NrHV-K protein location are listed. Aa numbering according to the consensus NrHV-K sequence. Cell culture-adapted substitutions from RHV-cc1 are marked with italics.

Amino acid position number	Protein	NrHV-Kcc1	NrHV-Kcc2	NrHV-Kcc3	NrHV-Kcc4
342	E1		Q342P		Q342P
586	E2	*L586S*	*L586S*	*L586S*	*L586S*
1757	NS4B	*S1757A*	*S1757A*	*S1757A*	*S1757A*
2373	NS5A	*T2373A*	*T2373A*	*T2373A*	*T2373A*
2398	NS5B			I2398V	I2398V

### Construction of the pNrHV-K clone and evaluation of infectivity *in vivo*

Next, we constructed a molecular consensus clone pNrHV-K, facilitating in-depth virus studies. To evaluate infectivity, we produced *in vitro* transcribed (IVT) RNA and intrahepatically inoculated two Lewis rats (r01 and r02). One-week post-infection (wpi), both rats displayed viremia, maintaining serum genome titers at approximately 10^8^ genome equivalents (GE)/mL throughout the experiment (Fig 1D). Deep sequencing analysis of the virus collected at one wpi revealed no non-synonymous mutations with frequencies ≥50% (Fig 1E). In samples collected at 8 wpi, a notable exception of a T1862M substitution was found at a frequency of 75.6% in rat r02 (Fig 1E).

### Adaptation of NrHV-K for replication *in vitro* and characterization of receptor use

**Fig 2:**
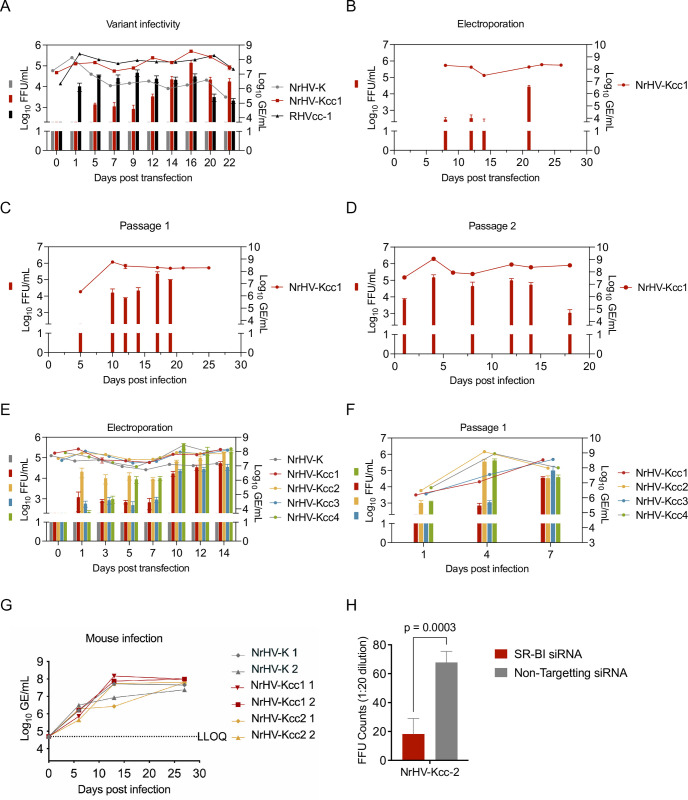
Development of an infectious cell culture system of NrHV-K. Genomic equivalent (GE) and focus forming unit (FFU) titers in McA-RH7777.hi [23] cell supernatants and mouse serum. (A) Viral titers following electroporation with *in vitro* transcripts (IVT) RNA of pNrHV-K, cell culture-adapted NrHV-K 1 (pNrHV-Kcc1) (Table 2), or cell culture-adapted RHV-rn1 (pRHVcc-1) as a control [22]. (B) Viral titers following second electroporation with IVT RNA of pNrHV-Kcc1. (C) Viral titers following passaging of virus quantified in panel (B) and collected 9 days post-transfection (dpt). (D) Viral titers following passaging of the virus quantified in panel (C) and collected 13 days post-infection. (E) NrHV titer following electroporation with IVT RNA encoding NrHV-K, NrHV-Kcc1, NrHV-Kcc2, NrHV-Kcc3, and NrHV-Kcc4 (Table 2). (F) Viral titers following passage of NrHV-Kcc1–4 quantified in panel (E) and collected 7 dpt. Error bars indicate the standard deviation of technical replicates for GE (n = 2) and FFU (n = 3) (A-F). (G) Serum virus GE titers in SCID mice following intraperitoneal inoculation with culture-derived NrHV-Kcc1, NrHV-Kcc2, and rat serum derived NrHV-K (r01, 1-week post-infection). The lower level of quantifications (LLOQ) for the assays were 2x10^2^ FFU/mL and 5x10^4^ GE/mL (A-G). (H) Infection of McA-RH7777.hi cells transfected with SR-BI-targeting or scrambled control siRNA. Error bars represent the standard deviation of technical replicates (n = 4) with a p-value determined by an unpaired t-test.

RNA transcripts from pNrHV-K were not readily infectious in vitro. Therefore, we introduced the cell culture-adaptive substitutions L586S, S1757A, and T2373A, previously identified in RHVcc-1 [22,23], creating a new construct named pNrHV-Kcc1 (Table 2). We subsequently electroporated McA-RH7777.hi cells with RNA transcripts of pNrHV-K, pNrHV-Kcc1, and pRHVcc-1 [22] for comparative analysis. Whereas NrHV-K transfection resulted in RNA replication but a lack of infectious particle production (Fig 2A), infectivity titers for NrHV-Kcc1 reached 1x10^3^ focus forming units (FFU)/mL at 5 days post-transfection (dpt) and further increased to 2x10^4^ and 3x10^5^ FFU/mL at 14 and 16 dpt, respectively. These values were comparable to those of RHVcc-1, with peak titers of 4x10^4^ FFU/mL at 1 dpt, dropping 10-fold by 20 dpt. Deep sequencing of NrHV-Kcc1 RNA recovered at 22 dpt identified a single dominant non-synonymous mutation in E1 encoding Q342P, present at 83.8% frequency.

In a second electroporation experiment with NrHV-Kcc1, peak infectivity titers reached 2x10^4^ FFU/mL at 21 dpt (Fig 2B). Serial passage increased viral fitness further, with first passage titers reaching 1x10^5^ FFU/mL at 17 days post-infection (dpi) and similar titers at 4 dpi in the second passage (Fig 2C and 2D). Deep sequencing of second passage NrHV-Kcc1 RNA at 12 dpi identified substitutions Q342P in E1 at >99.0% frequency and I2398V in NS5B at 75.5% frequency.

To explore the putative role of culture-acquired substitutions Q342P (E1) and I2398V (NS5B), we incorporated these into NrHV-Kcc1, resulting in three new constructs: pNrHV-Kcc2 (Q342P), pNrHV-Kcc3 (I2398V), and pNrHV-Kcc4 (Q342P and I2398V) (Table 2). Following electroporation of McA-RH7777.hi cells, the NrHV-Kcc1–4 constructs all produced infectious titers above the lower limit of quantification (LLOQ) at 1 dpt (Fig 2E) with NrHV-Kcc2 displaying the highest initial infectivity titers in the 10^4^ FFU/mL range. This increased to the 10^5^ FFU/mL range at 10–14 dpt. NrHV-Kcc1 and NrHV-Kcc3 exhibited identical infectivity with titers around 10^3^ FFU/mL during 1–7 dpt, followed by a tenfold increase by 10 dpt. NrHV-Kcc4 displayed the highest peak infectivity titers at 10 dpt, with titers exceeding 10^5^ FFU/mL.

We next passaged NrHV-Kcc1–4 in culture. NrHV-Kcc2 and NrHV-Kcc4 exhibited higher initial FFU titers during infection (Fig 2F). Deep sequencing of the viruses collected at 7 dpi determined that NrHV-Kcc2 and NrHV-Kcc4 remained stable, whereas NrHV-Kcc3 gained the Q342P E1 substitution (88.9%) and a second NS5B substitution, K2558R (50%). Interestingly, NrHV-Kcc1 once again acquired the Q342P (73.3%) E1 substitution, whereas the E2 adaptive substitution L586S had fully reverted to L586.

To determine if cell culture-adapted NrHV-K variants remained infectious *in vivo*, we inoculated two severe combined immunodeficiency (SCID) mice with culture-derived NrHV-Kcc1 (L586S, S1757A, and T2373A), two with culture-derived NrHV-Kcc2 (Q342P, L586S, S1757A, and T2373A), and as a control two with rat serum derived NrHV-K, using a dose of 1x10^5^ GE. All mice had detectable virus titers at 6 dpi and remained infectious at high titers until the experiment was terminated at 28 dpi (Fig 2G). Sanger sequencing of samples collected at 13 dpi confirmed that the cell culture-adaptive mutations were maintained *in vivo*, with NrHV-Kcc1 retaining L586S, S1757A, and T2373A, and NrHV-Kcc2 retaining Q342P, L586S, S1757A, and T2373A.

We previously established that RHV-rn1 relies heavily on the scavenger receptor class B type 1 (SR-B1) for efficient hepatocyte infection [22]. Following small interfering RNA (siRNA)-mediated SR-B1 knockdown in McA-RH7777.hi cells, we observed significantly reduced infection with NrHV-Kcc2, suggesting that NrHV-K likewise depended on SR-B1 for infection (Fig 2H)*.*

### Serial passage of NrHV-K in immunodeficient SCID mice does not increase viral fitness in wild-type (wt) mice

**Fig 3:**
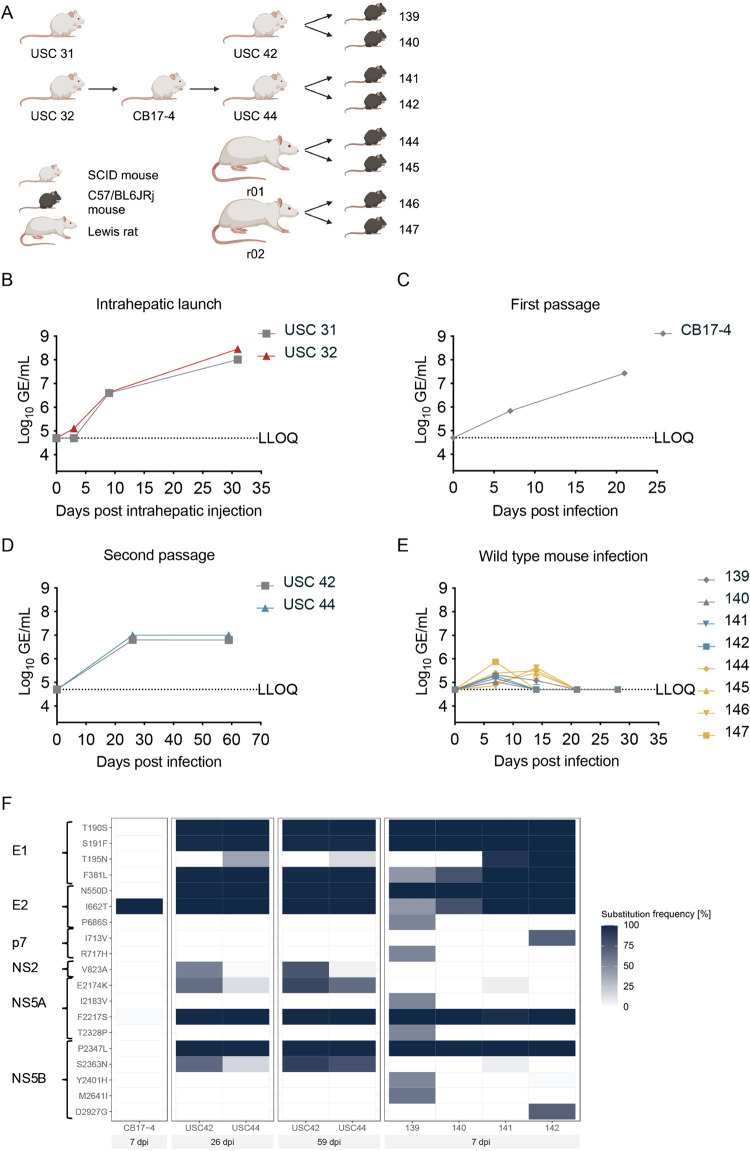
Infection of SCID mice and immunocompetent C57BL/6 mice with NrHV-K. (A) Experimental outline: Severe combined immunodeficiency (SCID) mice (USC31 and USC32) were inoculated intra-hepatically with *in vitro* transcribed RNA from pNrHV-K. At 7 days post-infection (dpi), serum from USC32 was inoculated intravenously (iv) into SCID mouse CB17–4. The second passage was performed by iv inoculation of USC42 and USC44 with serum from CB17–4 collected at 21 dpi. Subsequently, C57BL/6 mice were iv inoculated with mouse-adapted NrHV-K serum from USC42 (mice 139 and 140) and USC44 (mice 141 and 142) collected 56 dpi, and rat-derived NrHV-K serum from rats r01 (mice 144 and 145) and r02 (mice 146 and 147) collected 7 dpi. Created with BioRender. (B-E) Serum NrHV-K genomic equivalent (GE) titers during the passage of NrHV-K through SCID and C57BL/6 mice. (B) Viral titers following intra-hepatic inoculation of USC31 and USC32. (C) Viral titers following the first passage in CB17–4. (D) Viral titers following second passage in USC42 and USC44. (E) Viral titers following inoculation of C57BL/6 mice with mouse-passaged or wt NrHV-K. The lower level of quantification (LLOQ) was 5x10^4^ GE/mL. Error bars indicate the standard deviation of technical duplicates. (F) Virus evolution analysis of full-length NrHV-K coding sequence in SCID and C57BL/6 mice. Amino acid position and substitution frequency as % of total reads. The NrHV-K consensus sequence was used as reference genome. The dpi below the heat map indicates the sampling day (shaded area). The animal ID is listed above dpi information. The inclusion criteria for substitutions in this figure were a minimum substitution frequency of 50% in at least one sample.

Whereas RHV-rn1 can induce acute infection in mice, chronic infection has not been observed in wt mouse strains [20,22,29]. Passage of the NrHV-B isolate in immunodeficient mice resulted in prolonged acute infection in immunocompetent mice [20,24]. To assess infection of NrHV-K in mice, two CB17-SCID mice (USC31 and USC32) were intrahepatically inoculated with NrHV-K RNA transcripts (Fig 3A). Both mice developed viremia, reaching genome titers of ~10^8^ GE/mL by 31 dpi (Fig 3B). Subsequently, the virus collected from USC32 was passaged in naïve CB17-SCID mice, with a first passage in mouse CB17–4 resulting in peak titers of 3x10^7^ GE/mL at 21 dpi and second passages in two mice (USC42 and USC44) displaying viremia titers of approximately ~10^7^ GE/mL at 56 dpi (Fig 3C and 3D). To evaluate whether this virus had enhanced fitness in immunocompetent mice, eight C57BL/6 mice were infected with 10^5^ GE of mouse-passaged or wt (rat-derived) NrHV-K. Four mice were inoculated with serum collected at 56 dpi from SCID mouse USC42 (mice 139 and 140) or USC44 (mice 141 and 142). For comparison, four mice were inoculated with serum collected at 7 dpi from rat r01 (mice 144 and 145) or rat r02 (mice 146 and 147). Three mice infected with mouse-passaged NrHV-K cleared the virus by 14 dpi and the final mouse by 21 dpi and displayed slightly lower viral titers, ranging from 9x10^4^ to 2x10^5^ GE/mL, compared to those infected with rat-derived serum where titers ranged from 7x10^4^ to 7x10^5^ GE/mL, with one mouse clearing the virus by 14 dpi while the remaining three mice cleared the virus by 21 dpi (Fig 3E).

Deep sequencing of the first passage virus recovered from SCID mouse CB17–4 revealed the acquisition of an E2 substitution, I662T (Fig 3F). Following the second passage by 4 wpi, six additional substitutions (T190S, S191F, F381L, N550D, F2217S, and P2347L) emerged in both animals, USC42 and USC44, each exceeding 80% frequency. Upon inoculation into immunocompetent mice, distinct substitution patterns emerged. The virus sourced from USC42 evolved differently in recipient mice 139 and 140. In mouse 140, two signature substitutions, F381I and I662T, reverted. In mouse 139, six additional substitutions (P686S, R717H, I2183V, T2328P, Y2401H, M2641I) were acquired. In recipient mice 141 and 142 inoculated with USC44-derived virus, the T195N substitution, present as a minor variant (<50%) in the inoculum, became dominant (≥50%), as observed during mouse adaptations of RHV-rn1 [20]. In contrast, no dominant substitution could be detected in the mice infected with rat-derived NrHV-K (mice 144, 145, 146, and 147). The substitutions acquired during the passage of NrHV-K in SCID mice did thus not result in prolonged viremia within the immunocompetent mouse hosts, suggesting that a different adaptation strategy is required to achieve this goal.

### Mouse-passaged NrHV-K is attenuated in rats

**Fig 4:**
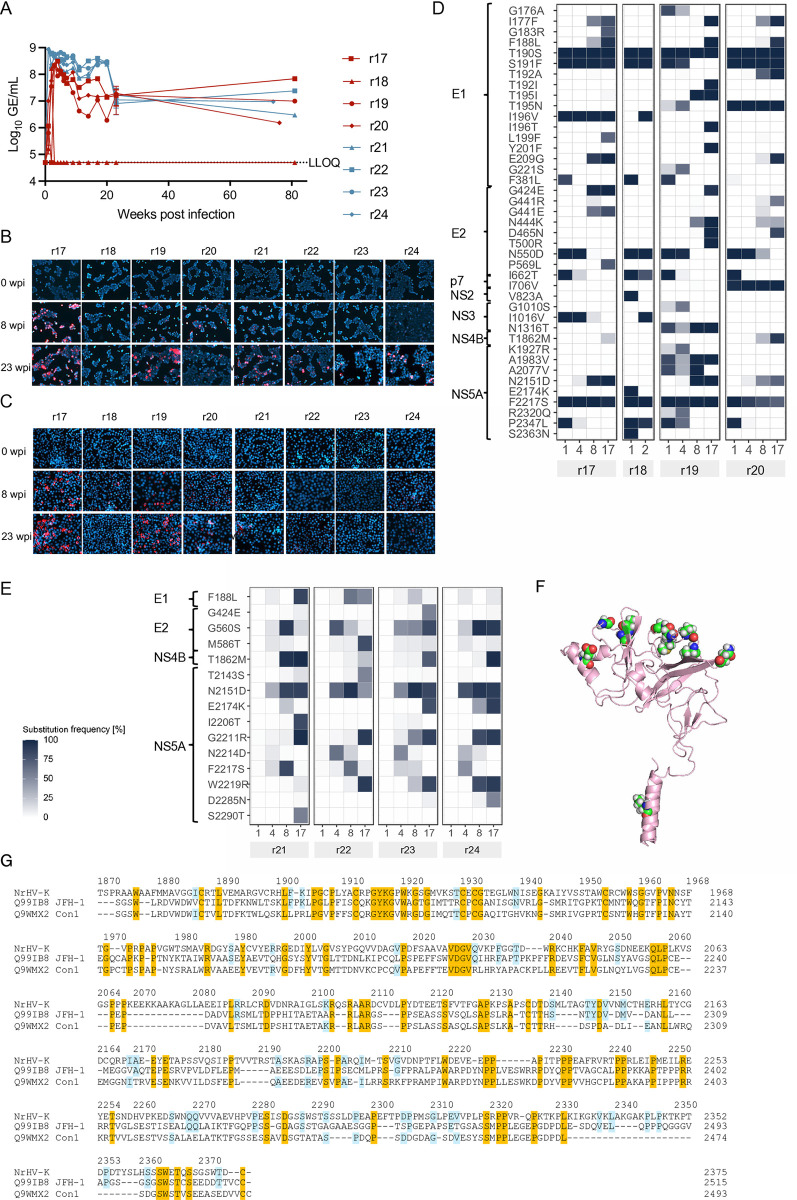
Evolution of NrHV-K infection and resulting humoral immune response in inbred Lewis rats. (A) Serum virus genomic equivalent (GE) titers following NrHV-K infection of naïve Lewis rats infected with 56 days post-infection SCID-mouse-derived NrHV-K serum from mouse USC 42 (r17 and r18) or mouse USC44 (r19 and r20). In parallel, rats were infected with NrHV-K positive serum sourced one-week post-infection (wpi) from rat r01 (r21 and r22) or r02 (r22 and r24). The lower level of quantification (LLOQ) was 5x10^4^ GE/mL. Error bars indicate the standard deviation of technical duplicates. (B, C) Detection of viral antigen reactive Abs in serum collected at 0-, 8-, and 23 wpi. (B) HEK-293T cells expressing recombinant RHV-rn1 E1-E2 antigen or (C) McA-RH7777.hi cells expressing an RHV-rn1 replicon [23] encoding NS3-NS5B. NrHV reactive Abs were visualized by Alexa fluor 594 immunostaining (red signal), and nuclear counterstained with Hoechst 33342 (blue). (D-E) Virus evolution analysis of full-length NrHV-K open reading frames in Lewis rats infected with (D) SCID-mouse-derived NrHV-K or (E) Lewis-rat-derived NrHV-K. Amino acid (aa) position and substitution frequency as % of total reads using NrHV-K consensus sequence as reference genome. Weeks-post infection and animal ID (shaded area) are shown below the heat map. The aa substitutions in the figure represent substitution frequencies >50% for at least one sample. (F) Ribbon diagram of predicted NrHV E2 structure [30]. Aa substitutions with >20% frequency in ≥3 animals are indicated as spheres (S5 Table). (G) Alignment of the NrHV-K NS5A aa sequence with that of HCV isolates Con1 (GenBank accession no. Q9WMX2) and JFH-1 (Q991B8). NrHV-K reference number according to the aa reference sequence. Conserved positions between all three sequences are highlighted with orange while positions matching NrHV and only one of the HCV isolates are highlighted in blue.

We next evaluated the impact of SCID mouse substitutions in the rat host by inoculating naïve Lewis rats with SCID mouse-derived NrHV-K. Two rats (r17 and r18) were inoculated with serum from USC42, and two additional rats (r19 and r20) were inoculated with serum from USC44. For comparison, two rats (r21 and r22) received serum from rat r01, and two (r23 and r24) received serum from rat r02. In rats infected with mouse-passaged NrHV-K, we observed dichotomous infection outcomes where viremia was initially detected one week after infection in all rats with titers of approximately 10^6^ GE/ml (Fig 4A). At 2 wpi, r19 and r20 had titer increases to ~10^8^ GE/ml and remained viremic throughout the experiment. Rats r17 and r18 displayed a different course of infection where the 2 wpi sample from r17 was below the LLOQ but subsequently increased to 10^8^ GE/mL the following week. In rat r18, the 2 wpi sample contained ~10^8^ GE/ml, as in rats r19 and r20. However, the titer decreased to below LLOQ the following week and remained below the detection limit until termination of the experiment. For r17, r19, and r20, we observed a 10-fold decrease in viral titers after 10 wpi. Rats r21-r24 infected with rat-derived NrHV-K developed persistent infection with titers around 10^8^ GE/mL through 20 weeks; after that, all four animals presented a 10-fold reduction, which remained until the termination of the experiment (Fig 4A).

Using recombinant RHV-rn1 E1-E2 [22] expressed in HEK 293T cells, we detected envelope-binding abs in the serum of rats r17, r19, and r20 as early as 8 wpi (Fig 4B), and more robust reactivity was observed at 23 wpi. In contrast, rats r21, r22, r23, and r24 demonstrated lower Ab reactivity than r17, r19, and r20. Only r21 had detectable Abs by 8 wpi. Further analysis at 23 wpi revealed moderate reactivity in r21, r22, r23, and r24. To assess antibody (Ab) responses against nonstructural proteins, we employed a sub-genomic replicon expressing the nonstructural NS3-NS5B proteins of RHV-rn1 in McA-RH7777.hi cells [23]. Consistent with the findings for E1-E2 reactivity, a similar response directed at nonstructural proteins was recorded in rats r17, r18, r21, and r20 at 8 wpi (Fig 4C). At 23 wpi, a more robust reactivity was observed, except for r18, where reactivity waned. Rats infected with rat-derived NrHV-K (r21, r22, r23, and r24) failed to demonstrate robust nonstructural Ab reactivity until 23 wpi, where r21 and r24 had detectable Abs.

Among the seven adaptive substitutions previously documented in SCID mice USC42 and USC44 (T190S, S191F, F381L, N550D, I662T, F2217S, and P2347L) only T190S and F2217S were retained in all four rats inoculated with the mouse-adapted virus (Figs 4D and S2), and S191F was retained in two of three persistently infected rats. In contrast, substitutions N550D and P2347L, along with F381L and I662T, reverted by 8 and 4 wpi, respectively. In rat r18 that cleared the virus within 3 wpi, only F381L had reverted prior to clearance. No ORF substitutions with ≥50% frequency was observed in the rats infected with rat-derived NrHV-K (r21, r22, r23, and r24) within 4 wpi (Fig 4E). Subsequently, N2151D in the NS5A region was identified after 4 wpi in all seven rats that did not clear infection and was consistently present throughout the experiment, exceeding 50% substitution frequency in all rats except r22 by 17 wpi (S2 and S3 Figs). No alterations within the UTR regions were detected at 8 wpi in the rats r21, r22, r23, and r24.

Analyses of substitutions developing beyond 17 weeks of infection until the end of the experiment (rats r17, r19, r20, 21, r22, r23, and r24) revealed that residues that became dominant (≥50%) were localized in the envelope proteins E1 and E2, and NS5A, as previously observed for RHV-rn1 [22]. For the glycoproteins E1 and E2, the majority of identified substitutions were located in the N-terminal domains (NTD) of both proteins (Fig 4D and 4E, and S5 Table). The structure of the E1 NTD (aa 176–212) could not be reliably predicted [30]. For E2, the mutations were clustered on the predicted membrane-facing surface of the protein (Fig 4F).

For rats infected with rat derived NrHV-K, in addition to N2151D, the NS5A substitutions G2211R and W2219R could be detected in 4 and 3 animals at >50%, respectively (Fig 4E). G2211R has previously been observed in an RHVcc-1 infected rat [22]. In rats infected with mouse-derived NrHV-K, aside from N2151D we did not observe any additional substitutions emerging in 3 or more animals (Fig 4D). Alignment of the NS5A aa sequence with the NS5A aa sequences of HCV isolates Con1 and JFH-1 suggest that the substitutions predominantly emerge in residues corresponding to the C-terminal of domain II and the low-complexity sequence II (Fig 4G) [31].

When examining rats r21, r22, r23, and r24, we detected no substitutions within previously identified MHC class II-restricted T-cell epitopes [32–34] but substitutions in two MHC class I-restricted T-cell epitopes (E1_191_ and E2_559_) [35,36], of which the G560S substitution in the E2_559_ epitope was observed in all four rats (S3 Fig). A greater number of emerging substitutions in putative T-cell epitopes were observed in rats r17, r19, and r20. Specifically, rat r19 was the only animal to exhibit substitutions in MHC class II-restricted T-cell epitopes; core_57_ and NS4B_1747_ [32,33] (S2 Fig). In MHC class I-restricted T-cell epitopes, substitutions emerged in three distinct locations (E1_191_, E1_224_, and NS3_1016_). E1_191_ was particularly noteworthy, as several substitutions developed within this epitope in all three rats.

### Induction of cross-neutralizing antibodies between two NrHV strains

**Fig 5:**
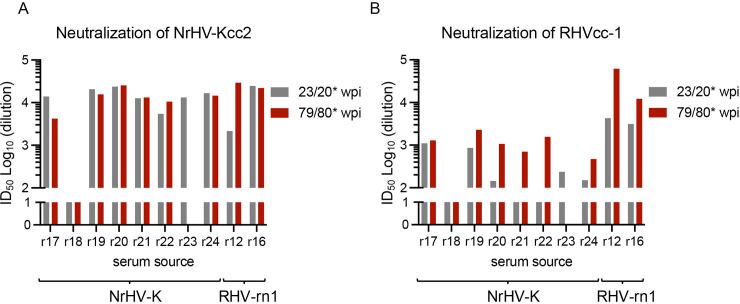
Temporal development of neutralizing antibodies in NrHV-K infected Lewis rats. (A, B) Serum dilution at which 50% infectivity titer reduction (ID_50_) is achieved upon NrHV-Kcc2 (A) or RHVcc-1 (B) infection. Serum samples obtained from rats r17, r18, r19, r20, r21, r22, r23^#^, and r24 (NrHV-K infected) and rats r12 and r16 (RHVcc-1 infected). For NrHV-K infected rats, serum was collected at 23 weeks post-infection (wpi) and at an average of 79 wpi. For RHVcc-1 infected rats, serum was collected at 20 wpi and 80 wpi, as represented with an asterisk. Lower limit of quantification (LLOQ) at 200-fold dilution. ^#^ r23 had met humane endpoint prior to the 81-week sampling.

We previously found that long-term RHV-rn1 infection in Lewis rats elicited nAbs emerging around 20 wpi [22]. Consistent with these findings, sera from NrHV-K infected rats displayed neutralizing activity in our infectious cell culture system at 23 wpi, with inhibitory dilution 50% (ID_50_) titers exceeding >10^3^ (Fig 5A). The sole exception was the serum from rat r18, which had cleared the virus.

Further assessment of cross-neutralization against the heterologous virus RHV-rn1 (RHVcc-1) revealed that sera from rats r17 and r19, collected at 23 wpi, exhibited detectable nAbs with titers approximating 10^3^ ID_50_ (Fig 5B). Sera collected at the same time point from rats r20, r23, and r24 showed detectable nAb titers but only marginally above the LLOQ, while no neutralization activity was detectable in sera from rats r18, r21, or r22 at 23 wpi. In contrast, serum samples procured from NrHV-K rats at an average of 79 wpi demonstrated increased neutralization titers, except for r18. Intriguingly, sera from two RHVcc-1 infected rats, r12 and r16 [22], sampled at both 20 and 80 wpi with nAbs against RHV-rn1 at titers of 10^3^ ID_50_, also neutralized the heterologous NrHV-K virus. Thus, we observed that infections with NrHV-K or RHV-rn1 elicit cross-neutralizing Abs.

### Spontaneous resolution of infection with mouse-adapted NrHV-K in rats

**Fig 6:**
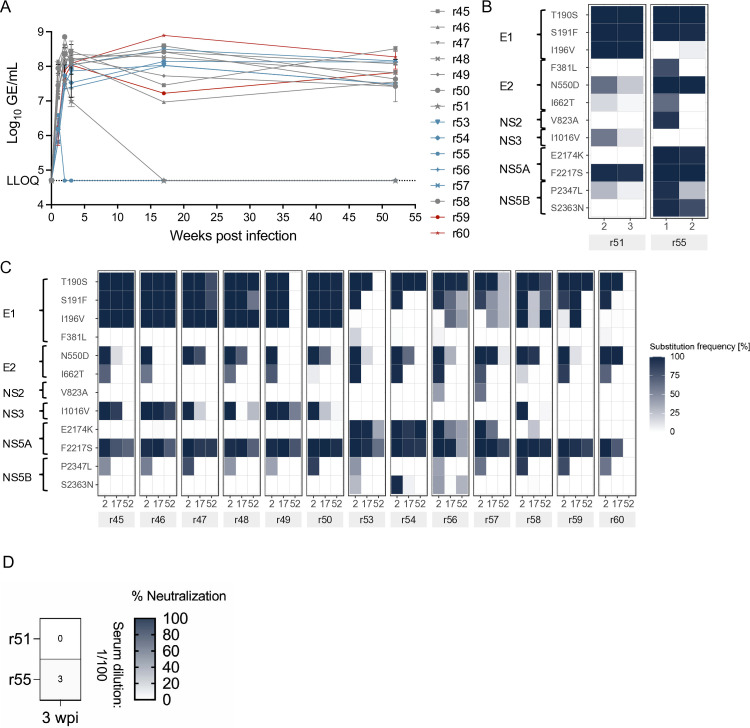
Assessment of chronicity rate for mouse adapted NrHV in Lewis rats. (A) NrHV viremia in Lewis rats following infection with NrHV-K positive serum from rat r17 week 1 (r45, r46, r47, r48, r49, r50, and r51 and r58), or rat r18 week 1 (r53, r54, r55, r56, and 57), or from mouse USC42 56 dpi (r59 and r60). The lower level of quantification (LLOQ) was 5x10^4^ genome equivalents (GE)/mL. Error bars indicate the standard deviation of technical duplicates. (B, C) NrHV-K evolution in Lewis rats that cleared the infection (B) or developed chronic infections (C). Amino acid position and substitution frequency as % of total reads. The NrHV-K consensus sequence was used as reference genome. The wpi and animal ID (shaded area) are shown below the heat map. The aa substitutions in the figure represent substitution frequencies >50% for at least one time point. (D) Neutralization capacity against NrHV-Kcc2 in serum collected 3 wpi from Lewis rats infected with 1 wpi rat-derived NrHV-K positive serum from rat r17 (r51) or rat r18 (r55). The heatmap shows the percentage of neutralization represented as the average of technical replicates (n = 3) at a 1:100 serum dilution.

To evaluate if we could reproduce the spontaneous clearance in rat r18 observed following inoculation with SCID mouse-passaged NrHV-K, we inoculated 15 additional naïve Lewis rats with rat serum from animals initially infected with SCID mouse-derived virus. Eight rats received serum derived from rat r17 and sampled at 1 wpi (r45-r51 and r58), and five rats received serum derived from rat r18 and sampled at 1 wpi (r53-r57). Finally, two rats received serum derived directly from SCID mouse USC42, collected at 56 dpi (r59 and r60). None of the rats inoculated with serum derived from mouse USC42 resolved the infection before experiment termination at 52 wpi (Fig 6A). In contrast, one rat from each of the r17 and r18 inoculations cleared the infection (r51 and r55, respectively). Combining the results, a total of 3 among 17 animals infected with virus stock originating from USC42 cleared NrHV-K infections, corresponding to a clearance rate of 18%. Deep sequencing analysis of viral samples collected before clearance did not reveal any substitutions exceeding >50% frequency for these rats that could explain the clearance (Fig 6B and 6C). The substitution at the potential glycosylation site at N550D was retained in both r51 and r55 prior to clearance. However, this substitution was also retained at 17 wpi in 6 of 13 rats that did not clear the virus. Additionally, no nAbs against cultured NrHV-K were detected for r51 or r55 at the time of clearance (Fig 6D). This finding aligns with the results observed in rat r18, where clearance occurred without detectable nAbs (Fig 5A).

## Discussion

The variability across HCV genotypes poses a substantial challenge to vaccine development, and ideally, a surrogate HCV model should thus mirror this antigenic diversity [37]. Among identified NrHV variants, experimental infectious systems were only available for closely related isolates of the RHV-rn1 variant [19,22,24], thus precluding assessment of immune responses against genetically diverse variants. In this study, we characterize NrHV-K, closely related to the originally characterized NYC-C12 [18] but divergent from RHV-rn1 [19]. We developed an infectious molecular clone demonstrating its potential for persistent infections in rats and SCID mice. Further, we successfully adapted it to *in vitro* conditions following incorporation of RHV-rn1 cell culture substitutions [22,23] and acquisition of an additional cell culture-adapted substitution, Q342P, previously observed for NrHV-B [24]. Developing an infectious clone resembling NYC-C12 allows a better reflection of NrHV variability and evolutionary dynamics.

As we conducted comprehensive sequencing of the NrHV-K ORF, the analysis revealed that similarly to HCV, NrHV-K exists as a heterogeneous population within the host. Compared to published variants, the genomic RNA sequence differed between NrHV-K and RHV-rn1 with 5.8%, a level of diversity less than that observed between different subtypes of HCV (>15% in the ORF) and more similar to the diversity observed between isolates of the same HCV subtype [38,39]. Since the number of reported NrHV isolates is limited, the degree of natural diversity remains unknown.

We demonstrated chronic NrHV-K infection in Lewis rats for a duration exceeding 81 weeks, where limited viral evolution was observed throughout the first four weeks of infection, except for the N2151D substitution in NS5A observed in all four rats infected with rat-derived NrHV-K (r21, r22, r23, and r24). This substitution has not previously been observed in rats infected with RHV-rn1 [22], and it persisted throughout the experiment. Additionally, following an average of 79 wpi, all four rats exhibited several additional substitutions (≥50%), most localized in the E1, E2, and NS5A regions of the genome. The same proteins were under intense selective pressure in RHV-rn1 infected rats, and it was recently shown that nAb pressure caused selection of escape mutations in the envelope proteins [22,40]. For NS5A, while the N2151D substitution has not been observed in RHV-rn1 infected rats, additional residues where previously observed substitutions occurred were 2211 and 2217, located in a suggested domain II [22]. At present, the basis for selection pressure in NS5A is unclear, and the substitutions are not in known T-cell epitopes [32–34,41].

Previously, serial passage and long-term adaptation of NrHV-B in immunodeficient NOD-Rag1^−/−^IL2Rγ^−/−^ mice led to adaptive substitutions prolonging viremia following infection of immunocompetent C57BL/6 mice [24]. Here, the passage of NrHV-K in SCID mice led to unaltered or shortened viremia in immunocompetent C57BL/6 mice compared to infection with the original NrHV-K, despite the acquisition of previously identified mouse-adaptive substitutions T190S, S191F, and N550D [20,24,29]. The shortened viremia of the SCID mouse-derived NrHV-K suggests a less fit virus in an immunocompetent host. This theory is further supported by the outcome in rats, where 17% of those infected with a mouse-adapted virus successfully cleared the infection. However, as the SCID strain employed here is derived from the BALB/c genetic background rather than C57BL/6, this difference could have influenced the results. It remains possible that challenging BALB/c mice with mouse-adapted NrHV-K may have demonstrated prolonged viremia, yet in the case of NrHV-B, increased fitness was observed even when challenging immunocompetent mice from a different genetic background [24].

A notable conundrum is the N550 substitution, which disrupts a confirmed glycosylation site [24] and potentially exposes the envelope further. Yet, inoculation with the mouse-adapted NrHV-B_SLIS_ variant, containing this N550S substitution, resulted in prolonged infections in immunocompetent mice [24]. While not fully understood, it is speculated that this mouse adaptation is tolerated due to the absence of nAbs selection pressure in mice, where viral clearance relies more heavily on T cells [20,24]. Overall, several substitutions from the serial passage were tolerated in rats, including the envelope substitutions T190S and S191F, and the NS5A substitution F2217S.

Our challenge of rats with mouse-passaged NrHV-K is the first report of a dichotomous infection outcome in inbred rats. Previously clearance has only been observed in outbred Holtzman rats using the RHV-rn1 isolate [22]. These results provide a unique opportunity to investigate viral variants with differential infection outcomes. The early viral clearance observed here suggests that innate immune responses may be involved, however, given the intact innate immunity in the SCID mice where these mouse-adapted variants were selected, replicating viruses must be capable of evading innate immune responses given the absent adaptive immunity. Because these findings are based on limited animal studies and a genetically heterogeneous challenge stock, further investigations are needed to identify if mutations are associated with viral clearance and understand their mechanistic impact on NrHV-K fitness and immune interactions.

Despite the high genetic identity between NrHV-K and RHV-rn1 compared to the diversity between HCV genotypes, we observed that limited differences in the E1 and E2 proteins (9 and 10 aa, respectively) substantially affected virus neutralization. Thus, sera from NrHV-K-infected rats could not efficiently neutralize RHVcc-1, early after nAbs emerged. However, we observed improved cross-neutralization in sera collected in the late stage of infection. Interestingly, sera from rats infected with RHVcc-1 did neutralize NrHV-Kcc2 at both early and late stages. Further, all NrHV-K-infected rats that remained viremic at 23 wpi developed nAbs, while only four out of nine rats administered RHV-rn1 or RHVcc-1 demonstrated neutralization at 20 wpi [22]. This suggests that NrHV-K may elicit a more robust nAb response. However, as NrHV-Kcc2 requires an additional E1 cell culture-adaptative substitution, Q342P, to produce infectious particles, we cannot rule out that the change in neutralization sensitivity is linked to this cell culture-adaptation and not pre-existing differences between the envelope proteins. While usage of NrHV-Kcc1 or NrHV-Kcc3, lacking the Q342P substitution, in theory could address this, low infectivity titers prevented their use in the neutralization assay. For HCV, it has been observed that single-point substitutions in the envelope proteins can drastically alter the sensitivity to nAbs [42]. Therefore, further studies are needed to investigate the differences in the immune response to these two NrHV variant types.

In summary, as NrHV becomes more widely used as a surrogate model for HCV, it is essential to expand the number of isolates further to approach the diversity encountered in HCV infection. We have initiated this effort by characterizing a novel NrHV isolate, NrHV-K, closely resembling the originally characterized NYC-C12 [18]. Developing an infectious molecular clone for NrHV-K and its successful *in vitro* adaptation constitutes a valuable resource in the repertoire of well-characterized NrHV isolates. Moreover, the availability of a molecular clone facilitates the rapid generation of chimeric viruses from newly identified NrHV isolates, paralleling the utility of JFH-1 as a chimera backbone in HCV research, thereby obviating the need to construct complete molecular clones for each isolate [43]. For instance, such chimeras could be readily generated and applied in neutralization studies by insertion of envelope proteins from different heterogeneous isolates into the replicating competent backbone of pNrHV-K [44]. Ultimately, a wider repertoire of NrHV isolates and subsequent development of infectious culture systems will promote the development of broad-spectrum vaccine candidates in the rat challenge model that can guide the generation of HCV vaccine concepts with similar broad efficacy.

## Materials and methods

### Ethics statement

All animal experiments were conducted under protocols 2017-15-0201-01288 and 2022-15-0201-01292, approved by the Danish Animal Experiments Inspectorate. Additionally, the study complied with the guidelines outlined in the Guide of Care and Use of Laboratory Animals published by the European Union (EU directive 2010/63/EU) and the ARRIVE (Animals in Research: Reporting In Vivo Experiments) standards. NrHV-K was isolated by infecting Lewis rats with a serum sample of a feral rat under protocol AR15–00116 (The Research Institute at Nationwide Children’s Hospital, USA).

### Sequence analysis of NrHV-K and construction of a full-length cDNA clone

Viral RNA was extracted from a serum sample collected from a Lewis rat experimentally infected with NrHV positive serum of a feral rat [18]. Complementary DNA (cDNA) encompassing the entire ORF and partial sequences of the 5’ and 3’ UTRs was subsequently synthesized by PCR [22] and cloned into a vector containing UTR sequences from RHV-rn1 [19] (GenBank number KX905133.1) and 10 clones (C1-C10, S1 Table) were Sanger sequenced (Macrogen Europe). Additionally, amplified ORFs were prepped utilizing the NEBNext DNA Ultra II kit (NEB) and deep sequenced using the Illumina MiSeq 500 or 150 PE V2 kit outlined in the S1 Text.

The 5’ UTR of the genomic RNA was subjected to rapid amplification of cDNA ends (RACE), and the 3’ UTR to homopolymeric tailing, cDNA synthesis, and PCR amplification with nested primer sets (S6 Table), cloned into pCR-XL-2-TOPO and Sanger sequenced (Macrogen Europe).

To complete the NrHV-K clone (pNrHV-K), the RHV-rn1 UTR sequences were mutagenized to the exact sequence of NrHV-K. Subsequently, cell culture-adapted NrHV-K (NrHV-Kcc1–4) (Table 2) was engineered from the pNrHV-K plasmid by site-specific megaprimer PCR mutagenesis [22–24] and electroporated into McA-RH7777.hi cells as outlined in the S1 Text.

### Laboratory animal experiments

Rat experiments were conducted in 10-week-old female LEWOrl/Rj rats (Janvier, Le Genest-Saint-Isle). Mouse experiments were conducted in 10–30-week-old immunodeficient CB-17/Icr-Prkdc^scid/scid^/Rj mice (Janvier) and C57BL/6JRj mice (Janvier). There was no randomization or blinding in the experimental setup.

Mice and rats were inoculated with IVT NrHV-K genomic RNA intrahepatically or by intravenous (iv) or intraperitoneal injection of viremic serum or cell-culture-derived supernatant (S7 Table). Details are in the S1 Text.

### Viral RNA quantification and sequencing

One-step TaqMan reverse transcription quantitative PCR quantified viral RNA titers in serum or culture supernatants [22]. Samples were processed for sequencing as described above in the sequence analysis section and the S1 Text.

### Prediction of MHC Class I-restricted T-cell epitopes

The SYFPEITHI algorithm was utilized to identify potential binding motifs for the MHC class I RT1-Al molecule specific to the Lewis rat [35,36]. Subsequently, putative epitopes scoring at least 21 out of a maximum of 28 points, representing the top 75% of scores, were considered in the analysis.

### Infectivity titration and neutralization assay

Infectivity titration and neutralization were performed as described [22] except that the virus (NrHV-Kcc2 or RHVcc-1) adjusted to 25,000 FFU/mL was incubated for 1 hour at 37˚C with serially diluted serum, heat-inactivated at 56˚C for 30 min, prior to seeding on host cells. Neutralization was calculated as outlined in the S1 Text.

### SR-BI knockdown

McA-RH7777.hi rat hepatoma cells were depleted of SR-BI by transfection with SR-BI-specific siRNA pools as previously described [22], except serially diluted cell culture-adapted NrHV-K virus stock was added to depleted McA-RH7777.hi cells and compared with cells receiving a non-specific scrambled siRNA pool.

### Expression of NrHV proteins

RHV-rn1 envelope proteins (E1-E2) and nonstructural proteins (NS3-NS5B) were expressed in HEK-293T and McA-RH7777.hi cells, respectively, following transfection with a DNA expression construct for E1-E2 and a self-replicating RNA encoding NS3-NS5B. The cells were fixed in methanol, and the presence of virus protein reactive Abs was assessed using rat serum, diluted 1:50. As a positive control for the detection of envelope proteins, a monoclonal mouse α-E2 Ab (clone 3G2) (S8 Table) [22] was used, while a polyclonal mouse α-NrHV IgG served to detect NS3 through NS5B. Bound rat Abs were detected with Alexa Fluor 594 goat α-rat IgG (A-11007; Thermo Fisher Scientific), whereas Alexa Fluor 594 goat α-mouse IgG (A-11005; Thermo Fisher Scientific) was used for the detection of mouse Abs in the positive control. Specific details are given in the S1 Text.

## Supporting information

S1 Fig Comparison of sequences of untranslated regions. (A, B) Detailed sequence of 5’ untranslated region (UTR) (A) and 3’UTR (B) of NrHV-K aligned to RHV-rn1. Nonidentical nucleotides (nt) relative to the published RHV-rn1 sequence (GenBank number KX905133.1) are highlighted in yellow, and microRNA 122 (miRNA-122) binding sites are indicated in red.
(PDF)

S2 Fig Comprehensive envelope evolution in Lewis rats infected with mouse-derived NrHV-K. Virus evolution analysis of full-length NrHV-K open reading frames in Lewis rats infected with mouse USC42-derived serum collected 56 days post-infection (dpi) (r17 and r18) or with mouse USC44-derived serum 56 dpi (r19 and r20). Amino acid (aa) position and substitution frequency as % of total reads. NrHV-K consensus sequence as reference genome. Weeks-post infection and animal ID (shaded area) are shown below the heat map. The aa substitutions in the figure represent substitution frequencies >50% for at least one sample. Substitutions in red indicate positions included in predicted MHC class I-restricted T-cell epitopes [35,36], and substitutions in green indicate positions included in experimentally determined MHC class II-restricted T-cell epitopes [32–34].
(PDF)

S3 Fig Comprehensive envelope evolution in Lewis rats infected with rat-derived NrHV-K. Virus evolution analysis of full-length NrHV-K open reading frames in Lewis rats infected with rat r1- (r21 and r22) or rat r2-derived (r23 and r24) serum collected one-week post-infection. Amino acid (aa) position and substitution frequency as % of total reads. NrHV-K consensus sequence as reference genome. Weeks-post infection and animal ID (shaded area) are shown below the heat map. The aa substitutions in the figure represent substitution frequencies >50% for at least one sample. Substitutions in red indicate positions included in predicted MHC class I-restricted T-cell epitopes [35,36].
(PDF)

S1 Table Overview of nucleotide (nt) sequence variants among NrHV-K Topo clones C1-C10. nt positions are numbered based on the full-length NrHV-K genome. The symbol “·” denotes identity with the consensus column, while “-“indicates absent nt. In the figure, “*” marks the location where extra nt are present that do not match any specific numbered position in the complete NRHV-K genome. In instances where the consensus cannot be determined, all possible nt are presented.
(DOCX)

S2 Table Overview of single-point substitutions identified through next-generation sequencing (NGS) analysis of the full-ORF of the original NrHV-K isolates from wild rats. The substitutions are listed in descending order of frequency. NrHV-K consensus sequence as reference genome. Only substitutions with a frequency ≥ 3.287% are included (median error (%) plus three times the standard deviation of the MiSeq Illumina platform [28]).
(DOCX)

S3 Table Overview of differences in amino acid (aa) sequence of NrHV isolates RHV-rn1, NrHV-A, NrHV-B, NrHV-K, and NYC-C12. The symbol “·” denotes identity with the RHV-rn1 sequence.
(DOCX)

S4 Table Diverging nucleotide count between NrHV-K and NYC-C12, or RHV-rn1, listed by individual proteins encoded by the entire ORF. (DOCX)

S5 Table Envelope protein mutations emerging with a substitution frequency >20% in at least 3 rats. (DOCX)

S6 Table List of oligonucleotide primer sequences. (DOCX)

S7 Table Overview of animals included in this study, with NrHV administration route, inoculum source, and dose. (DOCX)

S8 Table List of antibodies. (DOCX)

S9 Table Dataset contains Next-Generation Sequencing (NGS) data tracking somatic mutations in rats over multiple time points. Each row represents a specific nucleotide variant, detailing the reference (REF) and alternative (ALT) nucleotides, along with functional annotations such as functional class, codon change, and amino acid change (if applicable). Mutation frequencies are provided for individual rats across various time points. The sample identifiers follow the format rXX-Y, where rXX represents the rat ID and Y indicates the week of sample collection.
(XLSX)

S10 Table Dataset contains Next-Generation Sequencing (NGS) data tracking somatic mutations in mice over multiple time points. Each row represents a specific nucleotide variant, detailing the reference (REF) and alternative (ALT) nucleotides, along with functional annotations such as functional class, codon change, and amino acid change (if applicable). Mutation frequencies are provided for individual mice across various time points. The sample identifiers follow the format XXXX-Y, XXXX-YY or XXX-Y, where XXXX or XXX represents the mouse ID and YY or Y indicates the day of sample collection.
(XLSX)

S11 Table Dataset encompass viral titers expressed in genome equivalents (GE) or focus-forming units (FFU). Titers for each individual technical replicate, along with the averaged values, are presented together with timepoint of sample collection.
(XLSX)

S12 Table Dataset contains all neutralization data presented as either dilution at which 50% infectivity titer reduction (ID_50_) is achieved upon or as % neutralization. (XLSX)

S1 Text Detailed description of methods and materials. (DOCX)
