# Supplementary figures and images for: In vivo and in vitro recombinant systems of a novel variant demonstrate cross-reactive neutralization for the HCV model virus, Norway rat hepacivirus
Source: PLoS Pathog. 2025 Sep 25;21(9):e1013127. doi: 10.1371/journal.ppat.1013127 (PMC12782370; doi:10.1371/journal.ppat.1013127)

Supplementary figure 1

A

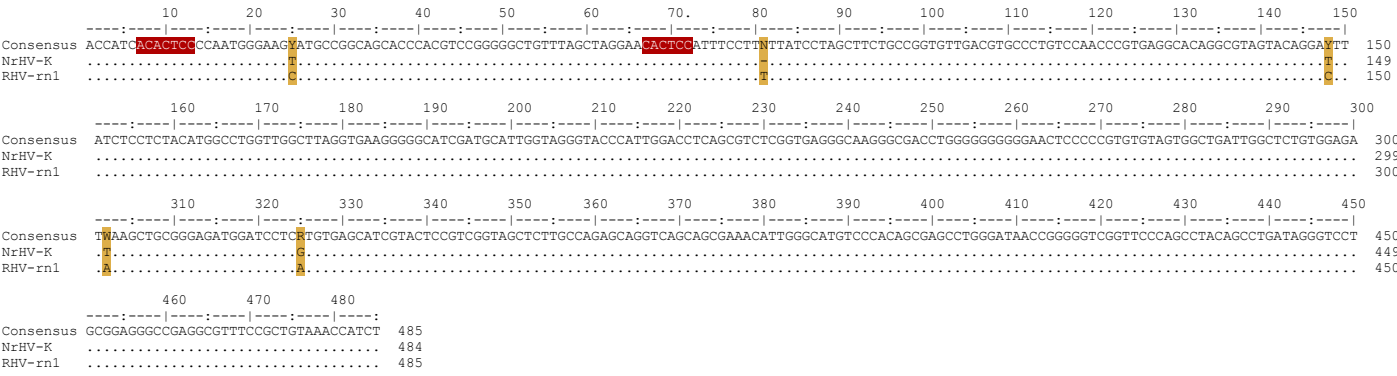

B

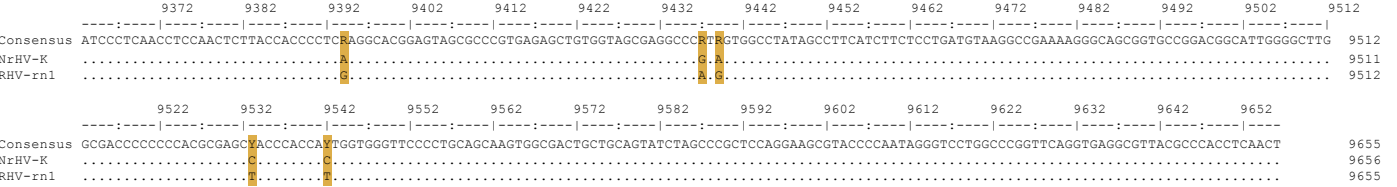

■ - miRNA-122 seed site  
■ - Difference

Supplement: S1 Fig — (A, B) Detailed sequence of 5’ untranslated region (UTR) (A) and 3’UTR (B) of NrHV-K aligned to RHV-rn1. Nonidentical nucleotides (nt) relative to the published RHV-rn1 sequence (GenBank number KX905133.1) are highlighted in yellow, and microRNA 122 (miRNA-122) binding sites are indicated in red. (PDF) [file ppat.1013127.s001.pdf]

Supplementary figure 2

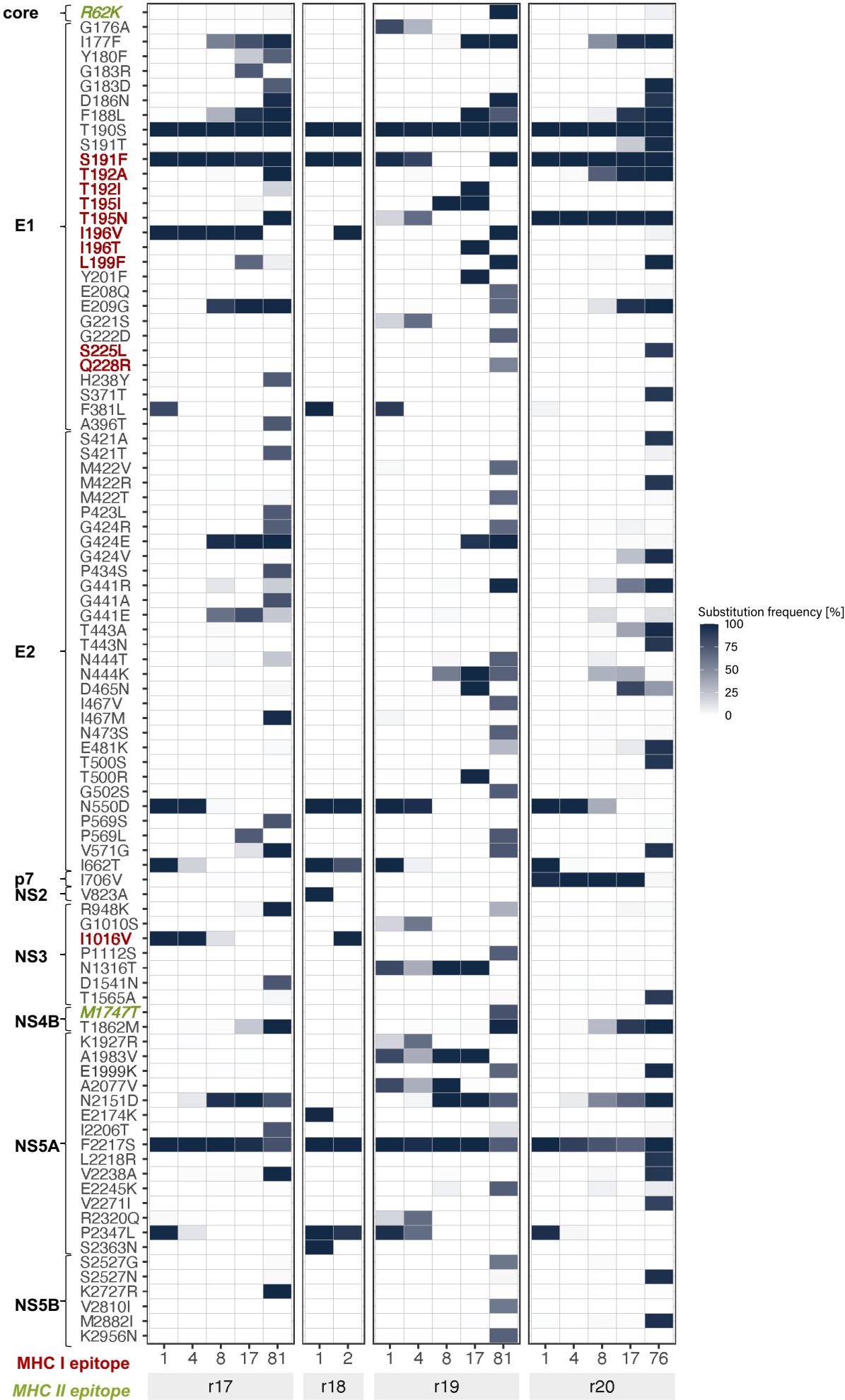

Supplement: S2 Fig — Virus evolution analysis of full-length NrHV-K open reading frames in Lewis rats infected with mouse USC42-derived serum collected 56 days post-infection (dpi) (r17 and r18) or with mouse USC44-derived serum 56 dpi (r19 and r20). Amino acid (aa) position and substitution frequency as % of total reads. NrHV-K consensus sequence as reference genome. Weeks-post infection and animal ID (shaded area) are shown below the heat map. The aa substitutions in the figure represent substitution frequencies >50% for at least one sample. Substitutions in red indicate positions included in predicted MHC class I-restricted T-cell epitopes [35,36], and substitutions in green indicate positions included in experimentally determined MHC class II-restricted T-cell epitopes [32–34]. (PDF) [file ppat.1013127.s002.pdf]

Supplementary figure 3

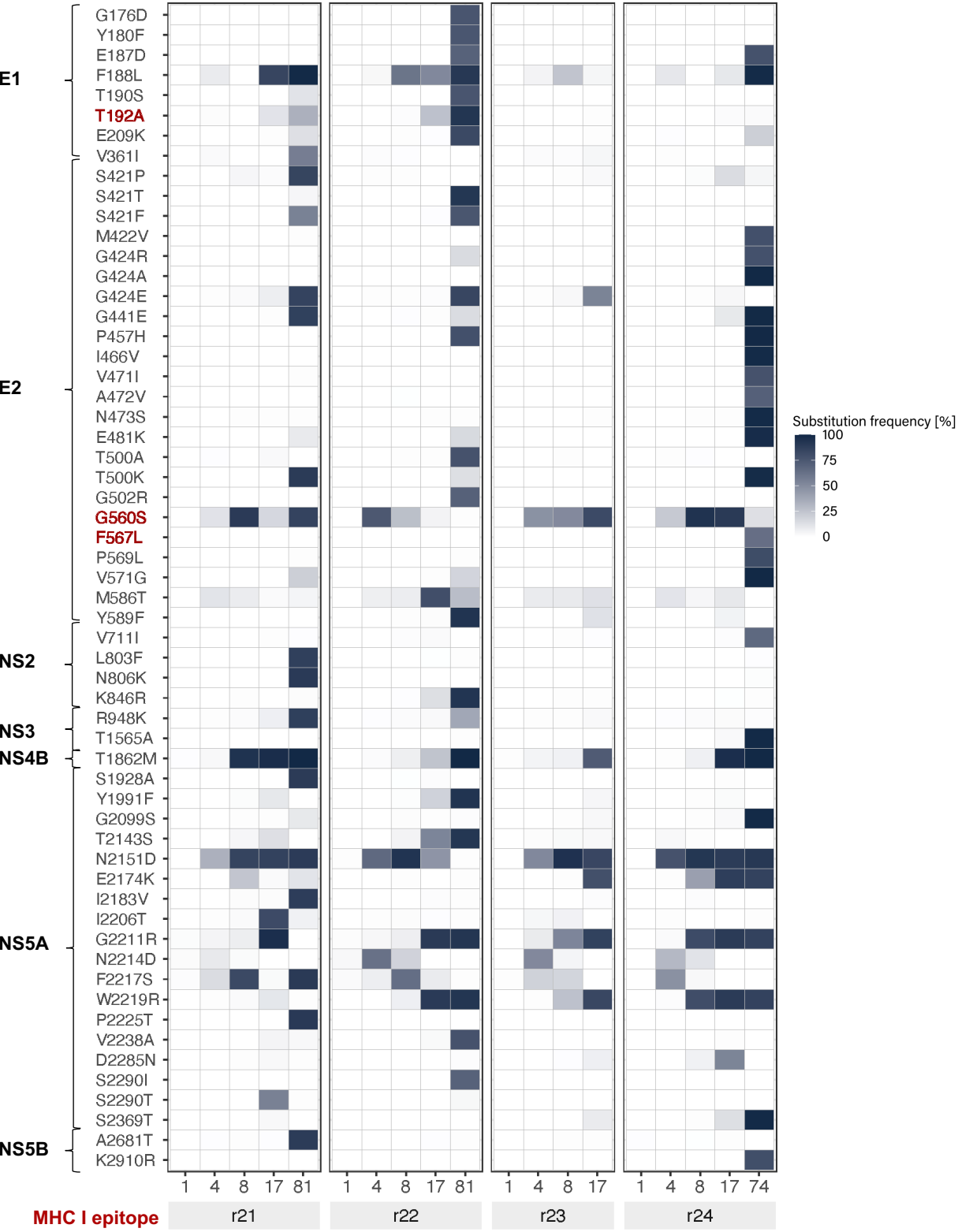

Supplement: S3 Fig — Virus evolution analysis of full-length NrHV-K open reading frames in Lewis rats infected with rat r1- (r21 and r22) or rat r2-derived (r23 and r24) serum collected one-week post-infection. Amino acid (aa) position and substitution frequency as % of total reads. NrHV-K consensus sequence as reference genome. Weeks-post infection and animal ID (shaded area) are shown below the heat map. The aa substitutions in the figure represent substitution frequencies >50% for at least one sample. Substitutions in red indicate positions included in predicted MHC class I-restricted T-cell epitopes [35,36]. (PDF) [file ppat.1013127.s003.pdf]
