# Supplementary material for: In vivo and in vitro recombinant systems of a novel variant demonstrate cross-reactive neutralization for the HCV model virus, Norway rat hepacivirus
Source: PLoS Pathog. 2025 Sep 25;21(9):e1013127. doi: 10.1371/journal.ppat.1013127 (PMC12782370; doi:10.1371/journal.ppat.1013127)
Supplement: S1 Table — The symbol “·” denotes identity with the consensus column, while “-“indicates absent nt. In the figure, “*” marks the location where extra nt are present that do not match any specific numbered position in the complete NRHV-K genome. In instances where the consensus cannot be determined, all possible nt are presented. (DOCX) [file ppat.1013127.s004.docx]

**S1 Table.**

| **NT Pos** | **Protein** | **Consensus** | **C1** | **C2** | **C3** | **C4** | **C5** | **C6** | **C7** | **C8** | **C9** | **C10** |
| --- | --- | --- | --- | --- | --- | --- | --- | --- | --- | --- | --- | --- |
| 528 | **core** | A | · | · | · | · | ⎯ | · | · | · | · | · |
| 529* |  | ⎯ | · | · | · | · | · | T | ⎯ | · | · | · |
| 952 |  | T | · | · | · | · | C | · | · | · | · | · |
| 1090 | **E1** | T | · | · | C | · | · | · | · | · | · | · |
| 1132 |  | C | · | · | · | · | · | T | · | · | · | · |
| 1228 |  | C | · | · | · | · | · | G | · | · | · | · |
| 1271 |  | A | · | · | · | · | · | · | · | · | G | · |
| 1494 |  | G | · | · | · | · | · | · | · | · | A | · |
| 1678 |  | T | · | · | · | · | · | · | · | · | · | C |
| 1681 |  | A | · | · | · | G | · | · | · | · | · | · |
| 1710 |  | T | · | · | · | C | · | · | · | · | · | · |
| 1779 | **E2** | A | · | · | G | · | · | · | · | · | · | · |
| 1804 |  | C | · | ·T | · | · | · | · | · | · | · | · |
| 1828 |  | T | · | · | · | · | · | · | C | · | · | · |
| 1847 |  | C | · | · | · | · | · | · | · | · | · | A |
| 2067 |  | G | · | · | · | · | · | · | · | · | · | A |
| 2197 |  | C | · | · | · | · | · | · | T | · | · | · |
| 2263 |  | C | · | · | · | · | · | · | · | · | T | · |
| 2395 |  | C | · | · | · | · | · | · | · | · | · | T |
| 2463 |  | T | · | · | · | · | · | C | · | · | · | · |
| 2551 |  | C/T | T | C | T | C | C | T | T | T | C | C |
| 2683 | **p7** | T | · | · | · | · | · | · | · | C | · | · |
| 2722 | **NS2** | C | · | · | · | · | · | · | · | T | · | · |
| 2857 |  | T | · | · | · | · | C | · | · | · | · | · |
| 2956 |  | T | · | · | · | G | · | · | · | · | · | · |
| 2992 |  | G | · | · | · | A | · | · | · | · | · | · |
| 3160 |  | T | · | · | · | · | · | · | C | · | · | · |
| 3181 |  | A/G | G | G | G | A | A | A | A | A | G | G |
| 3202 |  | T | A | · | · | · | · | · | · | · | · | · |
| 3464 | **NS3** | C | T | · | · | · | · | · | · | · | · | · |
| 3621 |  | T | · | · | · | C | · | · | · | · | · | · |
| 3626 |  | T | · | · | · | · | · | · | · | C | · | · |
| 3676 |  | A | · | · | G | · | · | · | · | · | · | · |
| 3787 |  | T | · | · | · | · | C | · | · | · | · | · |
| 3857 |  | G | · | · | · | · | · | · | · | A | · | · |
| 3955 |  | C | · | · | · | · | · | · | · | · | · | T |
| 4375 |  | C | · | · | · | T | · | · | · | · | · | · |
| 4795 |  | C | · | · | · | · | · | · | · | · | T | · |
| 4898 |  | G | · | · | · | · | A | · | · | · | · | · |
| 4983* |  | ⎯ | · | · | · | · | · | · | · | · | · | T |
| 5068 |  | G | · | · | · | A | · | · | · | · | · | · |
| 5077 |  | C | · | · | · | T | · | · | · | · | · | · |
| 5233 | **NS4A** | C | · | T | · | · | · | · | · | · | · | · |
| 5290 |  | C | · | · | · | · | · | · | · | · | · | T |
| 5677 | **NS4B** | C | · | · | · | T | · | T | · | T | · | T |
| 5755 |  | A | G | G | G | · | · | · | · | · | · | · |
| 5845 |  | C | · | · | · | · | · | T | · | · | · | · |
| 5881 |  | T | · | · | · | C | · | · | · | · | · | · |
| 5938 |  | A | · | · | · | · | G | · | · | · | · | · |
| 6069 |  | C | · | · | · | · | · | · | T | T | · | · |
| 6085 |  | C | · | · | · | · | · | T | · | · | · | · |
| 6160 | **NS5A** | A | · | · | · | · | · | · | · | G | · | · |
| 6229 |  | C | · | · | · | · | · | · | · | · | · | T |
| 6430 |  | G | · | T | · | · | · | · | · | · | · | · |
| 6442 |  | T | · | · | · | · | · | · | · | · | · | C |
| 6466 |  | C | T | · | · | · | · | · | · | · | · | · |
| 6547 |  | G | · | · | · | · | · | · | · | · | · | A |
| 6598 |  | T | · | · | C | · | · | · | · | · | · | · |
| 6619 |  | C | · | T | · | · | · | · | · | · | · | · |
| 7430 |  | C | · | · | T | · | · | · | · | · | · | · |
| 7618 | **NS5B** | G | · | · | · | · | · | A | · | · | · | · |
| 7826 |  | G | · | · | · | · | · | · | · | · | A | · |
| 7978 |  | G | · | · | · | · | · | · | · | T | · | · |
| 8167 |  | C | · | T | · | · | · | · | · | · | · | · |
| 8215 |  | G | · | A | · | · | · | · | · | · | · | · |
| 8596 |  | C | · | · | · | · | T | · | · | · | · | · |
| 8686 |  | A | · | · | · | · | · | · | G | · | · | · |
| 8884 |  | C | · | · | · | · | · | · | · | T | · | · |
| 9222 |  | T | · | C | · | · | · | · | · | · | · | · |
