# Supplementary material for: In vivo and in vitro recombinant systems of a novel variant demonstrate cross-reactive neutralization for the HCV model virus, Norway rat hepacivirus
Source: PLoS Pathog. 2025 Sep 25;21(9):e1013127. doi: 10.1371/journal.ppat.1013127 (PMC12782370; doi:10.1371/journal.ppat.1013127)
Supplement: S2 Table — The substitutions are listed in descending order of frequency. NrHV-K consensus sequence as reference genome. Only substitutions with a frequency ≥ 3.287% are included (median error (%) plus three times the standard deviation of the MiSeq Illumina platform [28]). (DOCX) [file ppat.1013127.s005.docx]

**S2 Table**.

| **nt position** | **Consensus** | **Alteration** | **Frequency** | **Functional class** | **Amino acid change** |
| --- | --- | --- | --- | --- | --- |
| 2551 | C | T | 42.29% | Synonymous | A689 |
| 3181 | A | G | 40.86% | Synonymous | V899 |
| 5755 | A | G | 26.32% | Synonymous | S1757 |
| 6069 | C | T | 16.87% | Non-synonymous | T1862M |
| 5677 | C | T | 16.18% | Synonymous | A1731 |
| 3955 | C | T | 12.04% | Synonymous | P1157 |
| 8944 | C | T | 8.02% | Synonymous | N2820 |
| 3327 | G | A | 5.40% | Non-synonymous | R948K |
| 1048 | T | C | 5.12% | Synonymous | F188 |
| 1132 | C | T | 4.78% | Synonymous | T216 |
