# Supplementary material for: In vivo and in vitro recombinant systems of a novel variant demonstrate cross-reactive neutralization for the HCV model virus, Norway rat hepacivirus
Source: PLoS Pathog. 2025 Sep 25;21(9):e1013127. doi: 10.1371/journal.ppat.1013127 (PMC12782370; doi:10.1371/journal.ppat.1013127)
Supplement: S3 Table — The symbol “·” denotes identity with the RHV-rn1 sequence. (DOCX) [file ppat.1013127.s006.docx]

**S3 Table**.

| **Aa Pos** | **Protein** | **RHV-rn1** | **NrHV-A** | **NrHV-B** | **NrHV-K** | **NYC-C12** |
| --- | --- | --- | --- | --- | --- | --- |
| 26 | **Core** | N | . | · | S | S |
| 179 | **E1** | P | . | S | . | . |
| 180 |  | Y | L | · | · | · |
| 183 |  | G | · | S | . | . |
| 184 |  | A | P | T | P | P |
| 188 |  | L | · | · | F | F |
| 192 |  | A | · | · | T | T |
| 196 |  | V | F | · | I | I |
| 199 |  | F | L | I | L | L |
| 207 |  | T | · | S | . | . |
| 208 |  | E | R | · | . | . |
| 212 |  | T | · | A | . | . |
| 221 |  | N | · | · | G | G |
| 223 |  | Q | · | · | · | R |
| 224 |  | R | · | · | Q | Q |
| 264 |  | T | · | · | A | A |
| 370 |  | V | · | · | A | A |
| 422 | **E2** | M | T | · | · | · |
| 424 |  | A | · | · | G | G |
| 435 |  | P | S | S | · | · |
| 441 |  | G | E | · | · | · |
| 458 |  | P | S | · | · | · |
| 459 |  | V | · | · | I | I |
| 477 |  | T | · | A | A | A |
| 481 |  | E | K | · | · | · |
| 500 |  | T | · | A | · | · |
| 502 |  | R | · | · | G | G |
| 505 |  | R | · | W | · | · |
| 518 |  | D | N | S | · | · |
| 552 |  | S | · | · | T | T |
| 569 |  | L | P | P | P | P |
| 570 |  | G | · | R | · | · |
| 571 |  | G | · | · | V | V |
| 572 |  | A | V | · | · | · |
| 586 |  | L | · | S | M | M |
| 609 |  | F | · | · | Y | Y |
| 653 |  | V | · | · | I | I |
| 711 | **p7** | I | · | · | V | V |
| 803 | **NS2** | F | · | · | L | L |
| 846 |  | R | · | · | K | K |
| 848 |  | M | · | · | L | L |
| 858 |  | S | · | · | F | F |
| 952 | **NS3** | N | · | · | S | S |
| 1122 |  | I | · | · | V | V |
| 1155 |  | R | · | · | K | K |
| 1397 |  | V | A | · | · | · |
| 1608 | **NS4A** | A | · | · | S | S |
| 1635 | **NS4B** | R | · | · | · | K |
| 1661 |  | I | · | V | · | · |
| 1862 |  | M | · | · | T | · |
| 2104 | **NS5A** | K | · | · | Q | Q |
| 2108 |  | V | · | · | A | A |
| 2151 |  | D | · | · | N | N |
| 2183 |  | V | · | · | I | I |
| 2207 |  | I | · | · | M | M |
| 2209 |  | K | · | · | S | S |
| 2213 |  | D | N | N | · | · |
| 2214 |  | N | · | K | · | · |
| 2216 |  | A | · | · | T | T |
| 2223 |  | E | · | V | · | · |
| 2224 |  | G | · | · | E | E |
| 2225 |  | Q | R | R | P | P |
| 2250 |  | L | · | · | I | I |
| 2269 |  | Q | · | · | · | R |
| 2290 |  | T | · | · | S | S |
| 2298 |  | D | · | · | E | E |
| 2315 |  | M | V | V | V | V |
| 2320 |  | Q | · | · | R | R |
| 2321 |  | S | · | · | P | P |
| 2328 |  | P | · | · | T | T |
| 2338 |  | R | · | · | K | K |
| 2357 |  | F | · | · | Y | Y |
| 2575 | **NS5B** | R | Q | · | · | · |
| 2579 |  | T | · | · | S | S |
| 2586 |  | K | · | N | · | · |
| 2699 |  | G | · | · | S | S |
| 2927 |  | N | · | · | D | D |
| 2928 |  | T | · | · | I | I |
| 2931 |  | D | · | · | E | E |
| 2935 |  | I | · | T | · | · |
