# Supplementary material for: In vivo and in vitro recombinant systems of a novel variant demonstrate cross-reactive neutralization for the HCV model virus, Norway rat hepacivirus
Source: PLoS Pathog. 2025 Sep 25;21(9):e1013127. doi: 10.1371/journal.ppat.1013127 (PMC12782370; doi:10.1371/journal.ppat.1013127)
Supplement: S4 Table — (DOCX) [file ppat.1013127.s007.docx]

**S4 Table.**

|  | **Diverging nucleotide count by protein between NrHV-K and isolates:** | |
| --- | --- | --- |
|  | **NYC-C12** | **RHV-rn1** |
| **Core** | 0 / 525 | 22 / 525 |
| **E1** | 2 / 726 | 51 / 726 |
| **E2** | 3 / 816 | 56 / 816 |
| **p7** | 0 / 162 | 11 /162 |
| **NS2** | 0 / 597 | 33 / 597 |
| **NS3** | 2 / 1,872 | 111 / 1,872 |
| **NS4A** | 0 / 162 | 9 / 162 |
| **NS4B** | 4 / 747 | 54 / 747 |
| **NS5A** | 3 / 1,518 | 87 / 1,518 |
| **NS5B** | 0 / 1,752 | 80 / 1,752 |
| **ORF** | 14 / 8,877 | 524 / 8,877 |
