# Supplementary material for: In vivo and in vitro recombinant systems of a novel variant demonstrate cross-reactive neutralization for the HCV model virus, Norway rat hepacivirus
Source: PLoS Pathog. 2025 Sep 25;21(9):e1013127. doi: 10.1371/journal.ppat.1013127 (PMC12782370; doi:10.1371/journal.ppat.1013127)
Supplement: S5 Table — (DOCX) [file ppat.1013127.s008.docx]

**S5 Table.**

| **Protein** | **Nucleotide position** | **Amino acid substitution** |
| --- | --- | --- |
| E1 | 1013 | I177F |
|  | 1031 | G183S |
|  | 1031 | G183R |
|  | 1031 | G183C |
|  | 1032 | G183D |
|  | 1032 | G183A |
|  | 1046 | F188I |
|  | 1046 | F188L |
|  | 1046 | F188V |
|  | 1053 | T190S |
|  | 1055 | S191T |
|  | 1055 | S191P |
|  | 1055 | S191A |
|  | 1056 | S191Y |
|  | 1056 | S191F |
|  | 1058 | T192P |
|  | 1058 | T192A |
|  | 1070 | I196L |
|  | 1070 | I196V |
|  | 1070 | I196F |
|  | 1079 | L199I |
|  | 1079 | L199F |
|  | 1109 | E209K |
|  | 1110 | E209A |
|  | 1110 | E209G |
|  | 1110 | E209V |
| E2 | 1745 | S421T |
|  | 1745 | S421P |
|  | 1745 | S421A |
|  | 1746 | S421Y |
|  | 1746 | S421C |
|  | 1746 | S421F |
|  | 1749 | M422K |
|  | 1749 | M422T |
|  | 1749 | M422R |
|  | 1754 | G424R |
|  | 1755 | G424E |
|  | 1755 | G424A |
|  | 1755 | G424V |
|  | 1806 | G441E |
|  | 1806 | G441A |
|  | 1816 | N444K |
|  | 1925 | E481K |
|  | 1983 | T500K |
|  | 1983 | T500R |
|  | 1983 | T500I |
|  | 2132 | N550D |
|  | 2190 | P569R |
|  | 2190 | P569L |
|  | 2196 | V571D |
|  | 2196 | V571A |
|  | 2196 | V571G |
|  | 2469 | I662T |
