# Supplementary material for: In vivo and in vitro recombinant systems of a novel variant demonstrate cross-reactive neutralization for the HCV model virus, Norway rat hepacivirus
Source: PLoS Pathog. 2025 Sep 25;21(9):e1013127. doi: 10.1371/journal.ppat.1013127 (PMC12782370; doi:10.1371/journal.ppat.1013127)
Supplement: S6 Table — (DOCX) [file ppat.1013127.s009.docx]

**S6 Table.**

| **No.** | **ID** | **Sequence** |
| --- | --- | --- |
| 1 | TS-O-00319 | GCTTCCTGGAGCGGGCTAGATACTG |
| 2 | TS-O-00316 | TGTCCCACAGCGAGCCTGGGATAAC |
| 3 | TS-O-00318 | CCAAGCCCCAATGCCGTCCGGCACCGCTGCCCTTTTCGG |
| 4 | TS-O-00259 | GATGGTTTACAGCGGAAACG |
| 5 | TSO | /5InvddT/GTCGCACGGTCCATCGCAGCAGTCACArGrGrG |
| 6 | TS-O-01515 | GTCGCACGGTCCATCGCAGCAGTC |
| 7 | TS-O-00854 | TCAGGCTGTAGGCTGGGAAC |
| 8 | TS-O-00178 | GGCCACGCGTCGACTAGTACTTTTTTTTTTTTTTTTTTTTVN |
| 9 | RHV-9020-F | AGCATACACGCCCAGGGAAA |
| 10 | RHV-full-F | ACCATCACACTCCCCAATGGG |
| 11 | TS-O-00258 | CCAGGCGTTGCATGATAACAC |
| 12 | RHV-full-R | AGTTGAGGTGGGCGTAACGC |
| 13 | TS-O-01674 | AATGTTTCGCTGCTGACC |
| 14 | TS-O-001719 | TTCGCTGCTGACCTGCTCTGGC |
| 15 | TS-O-001715 | CCAATGGGAAGCATGCCGG |
| 16 | TS-O-00404 | GGCGCTTGGTCGTGACAATG |
| 17 | TS-O-00554 | CGAGACGCTGAGGTCCAATG |
| 18 | TS-O-00313 | GGGCTGTTTAGCTAGGAACACTC |
| 19 | TS-O-00314 | TTTATCCTAGCTTCTGCCGGTGTTGACGTGCCCTGTCC |
| 20 | TS-O-00361 | AGGTGAAGGGGGCATCGATG |
| 21 | TS-O-1675 | CGCAGCTTTATCTCCACAGA (anti-sense) |
| 22 | TS-O-01171 | TACATGGCTAAGCAGTACGG (sense) |
| 23 | TS-O-01172 | GAGCGCGGCACCAATTCC (anti-sense) |
| 24 | TS-O-01173 | [6-FAM] CTCACGTACATGACGTATGGCATG [BHQ1a-6FAM] |
