# Supplementary material for: In vivo and in vitro recombinant systems of a novel variant demonstrate cross-reactive neutralization for the HCV model virus, Norway rat hepacivirus
Source: PLoS Pathog. 2025 Sep 25;21(9):e1013127. doi: 10.1371/journal.ppat.1013127 (PMC12782370; doi:10.1371/journal.ppat.1013127)
Supplement: S7 Table — (DOCX) [file ppat.1013127.s010.docx]

**S7 Table**.

| **Animal** | **Administration route** | **Source of NrHV** | **Dose** |
| --- | --- | --- | --- |
| r01 | Intrahepatic infection | IVT RNA pNrHV-K | 10 μg |
| r02 | Intrahepatic infection | IVT RNA pNrHV-K | 10 μg |
| r17 | Tail vein | USC42 serum collected 56 wpi | 10^5^ GE |
| r18 | Tail vein | USC42 serum collected 56 wpi | 10^5^ GE |
| r19 | Tail vein | USC44 serum collected 56 wpi | 10^5^ GE |
| r20 | Tail vein | USC44 serum collected 56 wpi | 10^5^ GE |
| r21 | Tail vein | r1 serum collected 1 wpi | 10^5^ GE |
| r22 | Tail vein | r1 serum collected 1 wpi | 10^5^ GE |
| r23 | Tail vein | r2 serum collected 1 wpi | 10^5^ GE |
| r24 | Tail vein | r2 serum collected 1 wpi | 10^5^ GE |
| r45 | Tail vein | r17 serum collected 1 wpi | 10^4^ GE |
| r46 | Tail vein | r17 serum collected 1 wpi | 10^4^ GE |
| r47 | Tail vein | r17 serum collected 1 wpi | 10^4^ GE |
| r48 | Tail vein | r17 serum collected 1 wpi | 10^4^ GE |
| r49 | Tail vein | r17 serum collected 1 wpi | 10^4^ GE |
| r50 | Tail vein | r17 serum collected 1 wpi | 10^4^ GE |
| r51 | Tail vein | USC42 serum collected 56 wpi | 10^4^ GE |
| r53 | Tail vein | r18 serum collected 1 wpi | 10^4^ GE |
| r54 | Tail vein | r18 serum collected 1 wpi | 10^4^ GE |
| r55 | Tail vein | r18 serum collected 1 wpi | 10^4^ GE |
| r56 | Tail vein | r18 serum collected 1 wpi | 10^4^ GE |
| r57 | Tail vein | r18 serum collected 1 wpi | 10^4^ GE |
| r58 | Tail vein | r17 serum collected 1 wpi | 10^4^ GE |
| r59 | Tail vein | USC42 serum collected 56 wpi | 10^4^ GE |
| r60 | Tail vein | USC42 serum collected 56 wpi | 10^4^ GE |
| USC32 | Intrahepatic infection | IVT RNA pNrHV-K | 10 μg |
| CB17-4 | Tail vein | USC32 serum collected 3 dpi | 1.3 x 10^3^ GE |
| USC42 | Tail vein | CB17-4 serum collected 21 dpi | 2.7 x 10^5^ GE |
| USC44 | Tail vein | CB17-4 serum collected 21 dpi | 2.7 x 10^5^ GE |
| 139 | Tail vein | USC42 serum collected 56 wpi | 10^5^ GE |
| 140 | Tail vein | USC42 serum collected 56 wpi | 10^5^ GE |
| 141 | Tail vein | USC44 serum collected 56 wpi | 10^5^ GE |
| 142 | Tail vein | USC44 serum collected 56 wpi | 10^5^ GE |
| 144 | Tail vein | r1 serum collected 1 wpi | 10^5^ GE |
| 145 | Tail vein | r1 serum collected 1 wpi | 10^5^ GE |
| 146 | Tail vein | r2 serum collected 1 wpi | 10^5^ GE |
| 147 | Tail vein | r2 serum collected 1 wpi | 10^5^ GE |
| NrHV-K 1 | Intraperitoneal | r1 serum collected 1 wpi | 10^5^ GE |
| NrHV-K 2 | Intraperitoneal | r1 serum collected 1 wpi | 10^5^ GE |
| NrHV-Kcc1 1 | Intraperitoneal | NrHV-Kcc1 | 10^5^ GE |
| NrHV-Kcc1 2 | Intraperitoneal | NrHV-Kcc1 | 10^5^ GE |
| NrHV-Kcc2 1 | Intraperitoneal | NrHV-Kcc2 | 10^5^ GE |
| NrHV-Kcc2 1 | Intraperitoneal | NrHV-Kcc2 | 10^5^ GE |
