# Supplementary material for: In vivo and in vitro recombinant systems of a novel variant demonstrate cross-reactive neutralization for the HCV model virus, Norway rat hepacivirus
Source: PLoS Pathog. 2025 Sep 25;21(9):e1013127. doi: 10.1371/journal.ppat.1013127 (PMC12782370; doi:10.1371/journal.ppat.1013127)
Supplement: S8 Table — (DOCX) [file ppat.1013127.s011.docx]

**S8 Table***.*

| **Name** | **Target** | **Conjugate** | **Provider** | **Catalog no.** | **Clone** | **Host species** | **Clonality** | **Isotype** | **Dilution** |
| --- | --- | --- | --- | --- | --- | --- | --- | --- | --- |
| α-RHV | RHV proteins | n.a. | Holmbeck, K (1) | n.a. | n.a. | Mouse | Polyclonal | IgG | 1:500 |
| Goat anti-Mouse IgG (H+L) Cross-Adsorbed Secondary Antibody | Mouse IgG | Alexa Fluor 594 | Thermo Fisher | A11005 | n.a. | Goat | Polyclonal | IgG | 1:500 |
| α-RHV-E2 antibody | RHV E2 | n.a. | Grakoui, A. (1) | n.a. | 3G2 | Mouse | Monoclonal | IgG | 1:50 |
| Goat anti-Rat IgG (H+L) Cross-Adsorbed Secondary Antibody | Rat IgG | Alexa Flour 594 | Thermo Fisher | A11007 | n.a. | Goat | Polyclonal | IgG | 1:500 |

## References

1. Wolfisberg R, Thorselius CE, Salinas E, Elrod E, Trivedi S, Nielsen L, et al. Neutralization and receptor use of infectious culture–derived rat hepacivirus as a model for HCV. Hepatology [Internet]. 2022 Nov 12;76(5):1506–19. Available from: https://journals.lww.com/10.1002/hep.32535
