# Supplementary material for: In vivo and in vitro recombinant systems of a novel variant demonstrate cross-reactive neutralization for the HCV model virus, Norway rat hepacivirus
Source: PLoS Pathog. 2025 Sep 25;21(9):e1013127. doi: 10.1371/journal.ppat.1013127 (PMC12782370; doi:10.1371/journal.ppat.1013127)
Supplement: S1 Text — (DOCX) [file ppat.1013127.s016.docx]

# S1 Text

# *In vivo* and *in vitro* recombinant systems of novel variant demonstrate cross-reactive neutralization for the HCV model, Norway rat hepacivirus

Caroline E. Thorselius^1^, Andreas Kok^1^, Raphael Wolfisberg^1^, Ulrik Fahnøe^1^, Matthew J. Kennedy^1^, Emma A. Lundsgaard^1^, Lotte Mikkelsen^1^, Mads K. Larsen^1^, Satyapramod Murthy^2^, Sheetal Trivedi^2^, Amit Kapoor^2^, Troels K. H. Scheel^1^, Kenn Holmbeck^1,#^, Jens Bukh^1,#,*^

^1^ Copenhagen Hepatitis C Program (CO-HEP), Department of Infectious Diseases, Copenhagen University Hospital, Hvidovre and Department of Immunology and Microbiology, Faculty of Health and Medical Sciences, University of Copenhagen, Denmark

^2^ Center for Vaccines and Immunity, Research Institute at Nationwide Children's Hospital, Columbus, Ohio, USA

#Authors contributed equally as senior authors

*Corresponding author:

Jens Bukh

Department of Immunology and Microbiology, University of Copenhagen

Blegdamsvej 3B, 07.13.84, DK-2200 Copenhagen N

e-mail: jbukh@sund.ku.dk

Phone: +45 23 41 89 69

## S1 Text: detailed methods

### Sequence analysis of NrHV-K and construction of a full-length cDNA clone (pNrHV-K)

For cloning and sequence determination of the complementary DNA (cDNA) covering the NrHV-K open reading frame (ORF), first strand was synthesized by incubation of RNA, with 0.1 µM RT primer #1 (S5 *Table*), 40 U of RNasin Plus RNase inhibitor (Promega), 0.5 mM dNTP mix (NEB) for 5 min at 70 ˚C then quenched on ice. The RNA template-primer mix was combined with 100 U of Maxima minus H reverse transcriptase (Thermo Fisher), 1X reaction buffer in a total of 20 µl and incubated for 120 min at 50˚C then subsequently 5 min at 85 ˚C. Subsequently, the RNA template was digested by incubation with 20 U of RNase H (Promega) for 20 min at 37 ˚C. Two µl of the resulting cDNA was amplified with 0.5 U of Q5 Hot start High Fidelity DNA polymerase (NEB) in 1X reaction buffer, 200 µM dNTP, 0.5 µM each primer #2 and #3 (S5 *Table*) in a 50 µL reaction supplemented with 1X High GC Enhancer using cycling parameters of initial denaturation at 98 ˚C for 30 s, followed by 37 cycles of 98 ˚C for 10 s, 65 ˚C for 20 s, and 72 ˚C for 8 min, and a final extension at 72 ˚C for 10 min. The amplified DNA was gel fractionated, excised, and recovered using a Zymoclean large fragment Gel DNA Recovery Kit (Zymo Research).

Amplified ORF’s were used for library preparation using a NEBNext DNA Ultra II kit (NEB) according to manufacturers’ instructions. The resulting fragmented indexed libraries were deep sequenced using either the Illumina MiSeq 500 or 150 PE V2 kit. Data was processed as previously described [1]. The consensus sequence generated from the original clonal analysis was used as reference genome for analysis. A cut-off value of 3.287% was set, corresponding to a previously determined median error value plus three standard deviations of the MiSeq Illlumina platform [2].

The NrHV-K ORFs were cloned into pRHV-rn1 [3] by megaprimer PCR mutagenesis using the NrHV-K ORF as megaprimer with cycling parameters: 98°C for 30 s, followed by 20 cycles of 98 °C for 10 s, 48 °C for 1 min, 72 °C for 20 min and a final extension at 72 °C for 20 min. The parental methylated pRHV-rn1 template DNA was digested with DpnI for 1 h at 37 °C (Thermo Fisher) and unrestricted unmethylated PCR-amplified plasmids were transformed into One Shot TOP10 Chemically Competent bacteria (Invitrogen) and plated on LB agar medium supplemented with 100 µg/mL ampicillin. Resistant colonies were picked and grown in 5mL LB medium supplemented with 100µg/mL ampicillin over-night, at 37 ℃, 300 rpm. Plasmid-DNA was purified using QIAprep Spin Miniprep Kit (Qiagen) according to manufacturer’s instructions. For plasmid medium size preparations, bacteria were grown in 150 mL of LB medium, supplemented with 100 µg/mL ampicillin for 72h, at 30 °C, 300 rpm. Plasmid DNA was extracted using the GenElute HP Plasmid Midiprep Kit (Sigma) according to the manufacturer’s instruction except 1.5 volumes of buffers (lysis, neutralization, and binding) were used in steps prior to DNA adsorption to the column. For *in vivo* experiments template DNA used in IVT RNA reactions, DNA was purified using the EndoFree Plasmid Maxi Kit (Qiagen).

For determination of terminal untranslated region (UTR) sequences, the 5’ end was resolved by template switch 5’ RACE. Briefly, 4 µL of uncapped RNA was incubated with 1 µM of RT primer #4 (S5 Table) and 1mM dNTP mix and incubated at 70 ˚C for 5 min, then, 4 ˚C and combined with template switch buffer (NEB), template switch primer #5 (S5 Table) and reverse transcriptase mix (NEB) and incubated at 42 ˚C for 90 min, then 85 ˚C for 5 min. 1 µL of the reverse transcribed DNA was amplified with Q5 Hot Start High-Fidelity Master Mix (2X) (NEB) and 0.5 µM primer #6 and #7 (S5 Table) in 25 µL by initial denaturation at 98˚C for 30 seconds followed by 5 cycles of denaturation for 10 s at 98 ˚C, 30 s of annealing/extension at 72 ˚C. The amplification was continued by 30 cycles of denaturation at 98 ˚C for 10 s annealing at 67 ˚C for 15 s and extension at 72 ˚C for 30 s with a final extension step of 5 min at 72 ˚C. The resulting PCR fragments were gel fractionated and cloned into pCR™-XL-2-TOPO™ according to the manufacturer’s instruction and ten clones C1-C10 were selected for sequence analysis (S1 *Table*).

For resolution of 3’ end sequences, purified RNA was tailed with polyA using 10 mM ATP, Yeast Poly(A) Polymerase (USB Affymetrix) and RNasin Plus RNase Inhibitor (Promega) at 37 ºC for 10 min. NrHV-K cDNA was then synthesized by preincubating the tailing reaction with adapter primer #8 (S5 Table) and 10 mM dNTP mix at 65 ºC for 5 min, then 48 ºC on hold, while adding the following RT mixture; Superscript III RT (Invitrogen), 0.1 M DTT and RNasin Plus RNase Inhibitor (Promega), before continuing the incubation by temperature escalation with a 48 ºC to 55 ºC gradient, followed by inactivation at 70 ºC for 15 min. Input RNA was degraded by incubation with RNase H and RNase T1 mix for 20 min at 37 ºC and the resulting cDNA was amplified with Q5 Hot Start High-Fidelity 2x Master Mix in 1^st^ step PCR using 10 µM abridged universal amplification primer (AUAP) and 10 µM primer #4 (S5 Table) and further reamplified in PCR 2^nd^ step, using AUAP and primer #9 (S5 Table). Identical cycling parameters were used in 1^st^ PCR and 2^nd^ PCR for both 5’ and 3’ determination; 98 °C for 1 min, followed by 40 cycles of 98 °C for 30 s, 52 °C for 40 s, 72 °C for 1 min, and a final extension at 72 °C for 5 min. The PCR products were purified from a 1% agarose gel, using the Zymoclean Gel DNA Recovery Kit (Zymo Research), cloned into pCR™-XL-2-TOPO™ according to the manufacturers’ instructions. All DNA constructs were sequenced by Sanger sequencing (Macrogen) and assembled using Sequencher or Geneious Prime (2023.0.2) by alignment using MAFFT into a consensus sequence using plurality base call.

Based on the consensus sequence deduced from multiple cloned 5’ and 3’ termini, the UTRs of the pRHV-rn1 construct containing the NrHV-K ORF was modified by sequential megaprimer amplification to incorporate the NrHV-K specific 5’ and 3’ UTR sequences. Briefly, primers #10 and #7 (S5 Table) for the 5’ UTR or #11 and #12 (S5 Table) for the 3’ UTR were used for PCR amplification of NrHV-K UTR consensus sequences using cycling conditions previously described for full ORF amplification. The resulting UTR fragments were gel fractionated and purified using the Zymoclean Gel DNA Recovery Kit (Zymo Research) and used as primers in a megaprimer mutagenesis reaction using the NrHV-K ORF containing pRHV-rn1 construct as template. The resulting pNrHV-K plasmid containing a T7 promotor flanking the 5’ UTR and a unique MluI restriction site sequence immediately after the 3’ UTR was verified by Sanger sequencing. A final consensus clone was selected for further work based on sequences from multiple plasmid mini preparations. Where ORF sequence information from Sanger sequences could not resolve a consensus base call, NGS sequence information from direct ORF sequencing of the serum derived cDNA was utilized to establish the base consensus identity.

For phylogenetic analysis, nucleotide (nt) sequences from relevant *hepaciviruses* covering the full-length open reading frame were aligned using MUSCLE version 5.1 in Geneious version 2023.2.1. using the Maximum Likelihood method and general time reversible model plus gamma model of nt substitution [4]. The bootstrap consensus tree was inferred from 100 replicates. The initial tree for the heuristic search was obtained by applying the neighbor-joint method to a matrix of pairwise distance estimates using the maximum composite likelihood approach [5,6].

Prediction of possible N-glycosylation sites in NrHV-K was done using NetNGlyc – 1.0 [7].

### Sequence determination of 5’ and 3’ UTR from infected Lewis rats r21-24

The 5’ UTR sequences of NrHV-K sampled from infected Lewis rats were resolved by 5’ RACE according to 5’ RACE System for Rapid Amplification of cDNA Ends, version 2.0 by Invitrogen. Briefly, RNA was denatured by incubation of 4 µL RNA with 1 µM of RT primer #4 (S6 Table) and 10mM dNTP mix for 5 min at 65 ˚, followed by subsequent quenching on ice. cDNA was generated from denatured RNA by adding 5x first-strand buffer (Invitrogen), 1 µL 0.1M DTT (Invitrogen), 0.5 µL RNasin Plus RNase inhibitor (Invitrogen), 2 µL SuperScript III RT (Invitrogen) and incubating for 30 min in a thermal gradient from 50 ˚C to 55 ˚C, increase 1˚C every 5 min, followed by an inactivation step for 15 min at 70 ˚C. The RNA template was digested by incubation with 0.5 µL of RNase mix (Invitrogen) for 20 min at 37 ˚C. cDNA was purified using SNAP column purification (Invitrogen) according to the manufacturer’s instructions.

For the tailing reaction, 16.5 µL purified cDNA was mixed with 5x tailing buffer (Invitrogen) and 2.5 µL 2mM dCTP, and incubated for 2 min at 94 ˚C, then left to chill on ice. Thereafter, 1 µL TdT (Invitrogen) was added and the tailing reaction was incubated for 10 min at 37 ˚C followed by heat activation for 10 min at 65 ˚C. dC-tailed cDNA was amplified with Q5 Hot Start High-Fidelity Master Mix (2X) (NEB), 10 µM Abridged Anchor Primer, and 0.5 µM primer #13 (S5 Table) in 25 µL by initial denaturation at 98˚C for 30 seconds followed by 40 cycles of denaturation for 30 s at 98 ˚C, 40 s of annealing at 52 ˚C, and 60 s extension at 72 ˚C, with a final extension step of 5 min at 72 ˚C. Thereafter, a nested amplification was performed by combining Q5 Hot Start High-Fidelity Master Mix (2X) (NEB), 10 µM AUAP (Invitrogen), and 0.5 µM primer #14 (S5 Table) in 25 µL, using the same cycling conditions as before. An internal stretch that was not captured by 5' RACE or ORF amplification, was amplified from previously generated cDNA, and amplified using primers #15 and #16 (S5 Table) with the same amplification and cycling conditions as above. DNA from the PCR reactions was column-purified using DNA clean and concentrator-5 (Zymo Research) according to the manufacturer’s instructions. Samples were sequenced by Sanger sequencing using primers #4, and #17-21 (S5 Table).

Sequencing of 3’ UTR were performed as previously described in section *Sequence analysis of NrHV-K and construction of a full-length cDNA clone (pNrHV-K)*, with the exception that the PCR products were directly subjected to Sanger sequencing instead of undergoing TOPO cloning as previously described.

### *In vitro* transcription

For generation of full-length NrHV-K RNA by *in vitro* transcription (IVT), 20 µg of pNrHV-K or pNrHV-Kcc1-4 was linearized by restriction with 40 Units of MluI-HF (NEB) for 2 h at 37 ℃ and cleaned on a DNA clean and concentrator-25 (Zymo Research). For *in vivo* studies endotoxin-free pNrHV-K was utilized. 5 µg of purified Mlu I linearized template was transcribed by incubation for 40 min at 37 ℃ with RiboMax Express T7 large scale reagents (Promega). The DNA template was removed by RQ1 RNase-free DNase (Promega) digestion for 30 min on ice. The RNA was further cleaned using a RNeasy mini kit (Qiagen) with on-column DNase treatment, eluted in nuclease-free water, and quantified with Qubit broad range RNA detection reagents (Invitrogen) prior to use.

### Electroporation of McA-rh7777.hi cells

McA-RH7777.hi rat hepatoma cells [8] were maintained in DMEM-Glutamax (Invitrogen) supplemented with 100 U/ml penicillin and 100 µg/ml streptomycin (Sigma) and 10% fetal bovine serum at 37 ℃, 5% CO_2_. On the day of electroporation, cells were trypsinized and pelleted by centrifugation at 700 rpm for 5 min at 4 ℃. The cells were washed in ice-cold PBS, pelleted, and re-suspended in Cytomix electroporation buffer (120 mM KCl, 0.15 mM CaCl_2_, 10 mM KPO_4_, 25 mM HEPES, 2 mM EGTA, and 5 mM MgCl_2_, adjusted to pH 7.6 with KOH) to a concentration of 1.5 x 10^7^ cells/mL [9]. 400 μL cell suspension was combined with 5 μg IVT RNA and electroporated in a 4 mm electrode gap cuvette using a GenePulser X Cell Electroporator (BioRad) set to exponential wave form protocol at 270 V, 975 μF, ∞ resistance. The cells were subsequently transferred to 10 cm cell culture dishes or T75 flask and maintained as described in this section with supernatant collection every 2-3 days for viral titration and sequencing.

### Laboratory animal experiments

All animals were housed in Innovive IVC caging containing wood chip bedding, shelters, nesting material, and biting sticks on a 12-hour light dark cycle. All experiments were conducted during the light cycle. Animals had access to food (SAFE D03, SAFE Complete Care Competence, Rosenheim, Germany) and water *ad libitum*.

RNA was inoculated by percutaneous intrahepatic injection under isoflurane anesthesia of 10 µg full-length IVT NrHV-K genomic RNA divided into two injections of 5 µg in separate hepatic locations. For inoculation with viral stocks, rats and mice were injected with NrHV-K through the tail vein or intraperitoneal under isoflurane anesthesia (S6 Table). Blood was sampled through tail vein cannulation (rats) or by facial venous puncture (mice) under isoflurane anesthesia according to national guidelines for blood sampling and collected in sterile tubes without anticoagulant unless otherwise specified until cell depletion.

### Viral RNA purification and qPCR quantification

For viral RNA purification, 25µl of rat or mouse serum was adjusted to 250µl with PBS and mixed with 750µl of TRIzol LS Reagent (Thermo Fisher) in a Phasemaker™ tube (Thermo Fisher) and incubated for 5 min at room temperature. Following addition of 200 μL chloroform (Sigma Aldrich), the sample was mixed, incubated 3 min at room temperature and centrifuged at 12,000 g for 15 min at 4˚C. The resulting aqueous phase was mixed with 450 μL of anhydrous ethanol, and RNA was purified on an RNA Clean & and Concentrator-5 column (Zymo Research) according to the manufacturer’s instructions.

Purified RNA was mixed with TaqMan™ Fast Virus 1-Step Master Mix (Thermo Fisher) and primers #22-24 (S5 Table) and quantified using a Cobas Z 480 light cycler analyzer (Roche). Cycling parameters used for viral genomic quantification as followed: 50 ℃ for 30 min, 95 ℃ for 5 min followed by 40 cycles of 95 ℃ for 15 s, 56 ℃ for 30 s, and 60 ℃ for 45 s. IVT pNrHV-K encoded RNA depleted for template DNA as described under IVT of RNA was serially diluted to generate a standard curve ranging from 10^9^-10^2^ GE, corresponding to a lower limit of quantification (LLOQ) to 5x10^4^ GE/mL.

### Infectivity titration and neutralization assay

For infectivity titration, 96-well plates were coated for 2 h at 37 °C with laminin (Sigma) at a concentration of 10 μg/mL and McA-RH7777.hi rat hepatoma cells were seeded at a density of 13,500 cells per well and incubated overnight under standard culture conditions as outlined in Supplementary materials S1 [8]. The next day, supernatants from infected cultures were serially diluted, added to the cells, and incubated for 48 h, then fixed with room temperature methanol for 5 min, and viral antigens were visualized by immunofluorescence staining using mouse α-NrHV IgG primary antibody (Ab) [3] and Alexa Flour 594 goat α-mouse IgG as secondary Ab (S7 Table). Imaging was performed at ×50 magnification using a Carl Zeiss Axio Vert.A1 microscope equipped with an Axiocam monochrome digital camera and Zen 2 Blue imaging software (Zeiss). The number of focus-forming units (FFU) was enumerated manually. One FFU was defined as one or more infected cells separated by at least two non-infected cells from any other FFU. Data points represent means ± standard deviations (SD) of triplicate counts.

For serum neutralization, two-fold serially diluted sera heat inactivated at 56 ℃ for 30 min were mixed with virus (NrHV-Kcc2 or RHVcc-1) adjusted to 25,000 FFU/mL and incubated at 37 ℃ for 1 h. Thereafter, the virus/serum mixture was incubated with McA-RH7777.hi rat hepatoma cells pre-seeded in laminin-coated 96-well plates for 4 h at 37 ℃, then removed, and the cells were washed with PBS and incubated for another 44 h in growth medium before methanol fixation as described previously in this section. Neutralization was calculated as percentage reduction in FFU count relative to a non-immune control. Fifty percent of maximal inhibitory dilution (ID_50_) values were calculated using the AAT BioQuest calculator [10].

### Expression of NrHV proteins

NrHV proteins were expressed as previously described [3,8]. Briefly, for E1/E2 expression HEK-293T cells were plated in poly-L-lysine coated 96-well plates, and for the expression of nonstructural proteins, McA-RH7777.hi cells seeded in laminin-coated 96-well plates and incubated overnight at 37 ℃ with 5% CO_2_. The following day, 5 μg of DNA encoding the most C-terminal 56 residues of core followed by E1 and E2 (RHV-rn1 polyprotein residues 120-689) was transfected into HEK-293T cells using Lipofectamine® 2000 (Thermo Fisher). For transfection of McA-RH7777.hi cells 2.5 μg of IVT RNA of a subgenomic replicon encoding encephalomyocarditis virus IRES followed by the RHV-rn1 nonstructural proteins NS3-NS5B [8] was utilized.

## References

1. Billerbeck E, Wolfisberg R, Fahnøe U, Xiao JW, Quirk C, Luna JM, et al. Mouse models of acute and chronic hepacivirus infection. Science. 2017;357: 204–208. doi:10.1126/science.aal1962

2. Stoler N, Nekrutenko A. Sequencing error profiles of Illumina sequencing instruments. NAR Genom Bioinform. 2021;3. doi:10.1093/nargab/lqab019

3. Wolfisberg R, Thorselius CE, Salinas E, Elrod E, Trivedi S, Nielsen L, et al. Neutralization and receptor use of infectious culture–derived rat hepacivirus as a model for HCV. Hepatology. 2022;76: 1506–1519. doi:10.1002/hep.32535

4. Nei M, Kumar S. Molecular Evolution and Phylogenetics. New York, US: Oxford University Press; 2000.

5. Tamura K, Stecher G, Kumar S. MEGA11: Molecular Evolutionary Genetics Analysis Version 11. Mol Biol Evol. 2021;38: 3022–3027. doi:10.1093/molbev/msab120

6. Stecher G, Tamura K, Kumar S. Molecular Evolutionary Genetics Analysis (MEGA) for macOS. Mol Biol Evol. 2020;37: 1237–1239. doi:10.1093/molbev/msz312

7. Gupta R, Brunak S. Prediction of glycosylation across the human proteome and the correlation to protein function. Pac Symp Biocomput. 2002; 310–22. Available: http://www.ncbi.nlm.nih.gov/pubmed/11928486

8. Wolfisberg R, Holmbeck K, Nielsen L, Kapoor A, Rice CM, Bukh J, et al. Replicons of a Rodent Hepatitis C Model Virus Permit Selection of Highly Permissive Cells. James Ou J-H, editor. J Virol. 2019;93: 733–752. doi:10.1128/JVI.00733-19

9. van den Hoff MJ, Moorman AF, Lamers WH. Electroporation in “intracellular” buffer increases cell survival. Nucleic Acids Res. 1992;20: 2902. doi:10.1093/nar/20.11.2902

10. AAT Bioquest Inc. Quest Graph^TM^ IC50 Calculator. In: AAT Bioquest [Internet]. [cited 19 Mar 2024]. Available: https://www.aatbio.com/tools/ic50-calculator
